# A Phase 1 Human Immunodeficiency Virus Vaccine Trial for Cross-Profiling the Kinetics of Serum and Mucosal Antibody Responses to CN54gp140 Modulated by Two Homologous Prime-Boost Vaccine Regimens

**DOI:** 10.3389/fimmu.2017.00595

**Published:** 2017-05-24

**Authors:** Sven Kratochvil, Paul F. McKay, Jakub T. Kopycinski, Cynthia Bishop, Peter John Hayes, Luke Muir, Christopher L. Pinder, Deniz Cizmeci, Deborah King, Yoann Aldon, Bruce D. Wines, P. Mark Hogarth, Amy W. Chung, Stephen J. Kent, Kathrin Held, Christof Geldmacher, Len Dally, Nelson S. Santos, Tom Cole, Jill Gilmour, Sarah Fidler, Robin J. Shattock

**Affiliations:** ^1^Imperial College London, Medicine, London, UK; ^2^Nuffield, University of Oxford, Medicine, Oxford, UK; ^3^Flow Cytometry Core Facility, Biomedical Research Centre, Guy’s Hospital, London, UK; ^4^Human Immunology Laboratory, IAVI, London, UK; ^5^Burnet Institute, Melbourne, VIC, Australia; ^6^Department of Microbiology and Immunology, Peter Doherty Institute for Infection and Immunity, University of Melbourne, Melbourne, VIC, Australia; ^7^ARC Centre of Excellence in Convergent Bio-Nano Science and Technology, University of Melbourne, Melbourne, VIC, Australia; ^8^Melbourne Sexual Health Centre, Department of Infectious Diseases, Alfred Health, Central Clinical School, Monash University, Melbourne, VIC, Australia; ^9^Division of Infectious Diseases and Tropical Medicine, Medical Center of the University of Munich (LMU), Munich, Germany; ^10^Emmes Corporation, Rockville, MD, USA; ^11^NIHR/Wellcome Trust Imperial Clinical Research Facility Hammersmith Hospital, Imperial College London, London, UK

**Keywords:** IgG subclasses, vaccine interval, human immunodeficiency virus vaccine, adjuvant, homologous prime-boost strategy, human immunodeficiency virus envelope protein, mucosal compartment

## Abstract

A key aspect to finding an efficacious human immunodeficiency virus (HIV) vaccine is the optimization of vaccine schedules that can mediate the efficient maturation of protective immune responses. In the present study, we investigated the effect of alternate booster regimens on the immune responses to a candidate HIV-1 clade C CN54gp140 envelope protein, which was coadministered with the TLR4-agonist glucopyranosyl lipid A-aqueous formulation. Twelve study participants received a common three-dose intramuscular priming series followed by a final booster at either 6 or 12 months. The two homologous prime-boost regimens were well tolerated and induced CN54gp140-specific responses that were observed in both the systemic and mucosal compartments. Levels of vaccine-induced IgG-subclass antibodies correlated significantly with FcγR engagement, and both vaccine regimens were associated with strikingly similar patterns in antibody titer and FcγR-binding profiles. In both groups, identical changes in the antigen (Ag)-specific IgG-subclass fingerprint, leading to a decrease in IgG1 and an increase in IgG4 levels, were modulated by booster injections. Here, the dissection of immune profiles further supports the notion that prime-boost strategies are essential for the induction of diverse Ag-specific HIV-1 responses. The results reported here clearly demonstrate that identical responses were effectively and safely induced by both vaccine regimens, indicating that an accelerated 6-month regimen could be employed for the rapid induction of immune responses against CN54gp140 with no apparent impact on the overall quality of the induced immune response. (This study has been registered at http://ClinicalTrials.gov under registration no. NCT01966900.)

## Introduction

The human immunodeficiency virus (HIV) continues to infect nearly two million people every year with one million AIDS-related deaths occurring annually (1). A critical step toward the effective control of the HIV epidemic is the development of an efficacious HIV vaccine, but despite concerted efforts spanning over 30 years, an effective AIDS vaccine remains elusive (2, 3).

Two phase III trials (VAX 004/003), using monomeric AIDSVAX clade B/E gp120 proteins as immunogens, failed to show efficacy in reducing HIV acquisition (4, 5), despite induction of high antibody binding titers following an autologous prime-boost regime. In contrast, the Thai phase III vaccine regimen (RV144), comprising two priming injections of a recombinant canarypox vector vaccine (ALVAC-HIV) followed by two booster injections of ALVAC-HIV combined with a recombinant gp120 subunit vaccine (AIDSVAX B/E), demonstrated a modest efficacy of 31.2% against HIV acquisition at 42 months after vaccination (6). Notably, IgG antibodies against the variable regions 1 and 2 (V1–V2) of HIV-1 envelope proteins correlated with a decreased risk of HIV acquisition (7) and *post hoc* analysis of RV144 estimated efficacy at 6 months after vaccination to be 60.5%, indicating an early protective vaccine effect that declines over time (8).

More recent evidence suggests that antigen (Ag)-specific immunoglobulin (Ig) diversification (9) may represent another important criterion for preventing HIV infection. An immune-correlates analysis of the RV144 trial identified that vaccine-induced Env V1–V2 IgG3 levels correlated with a decreased risk of HIV-1 infection and declined in line with the observed vaccine efficacy (10). It has long been recognized that different IgG subclasses (IgG1–4) have different effector functions, both in terms of triggering FcγR-expressing cells, resulting in antibody-dependent cellular phagocytosis (ADCP) or antibody-dependent cell-mediated cytotoxicity (ADCC), and activating complement. IgG1, IgG3, and IgG4 are commonly associated with B cell-mediated responses to protein Ags, while heavily glycosylated Ags are often associated with IgG2 responses (11). Correlation of V1–V2 IgG3 levels with reduced risk of acquisition in the RV144 trial has driven interest in the induction of IgG3 antibodies. IgG3 is characterized by an elongated hinge region of up to 62 amino acids (11) and has been associated with high Fc gamma receptor (FcγR) binding of IgG3 immune complexes (12). Furthermore, the extended hinge region equips IgG3 with a high degree of conformational flexibility, suggesting an improved potential for penetrating the protective HIV glycan shield, which could be crucial for preventing HIV immune evasion.

Although encouraging, the modest and transient efficacy observed in RV144 trial highlights the need for greater understanding of the parameters modulating the serological fingerprint of vaccine-induced HIV antibodies and their capacity for driving Fc-mediated effector functions, such as ADCC and ADCP, following vaccination (13, 14). While ADCC is associated with the crosslinking of FcγRIIIa (CD16a), ADCP is mediated primarily through FcγRIIa (CD32a). These known interactions provide the framework for a recently published assay, utilizing FcγR ectodomain dimers to probe for Fc-dependent functions (15, 16).

In addition to the HIV immunogen (vaccine vector and vaccine dosage) and choice of adjuvant, the effective induction of desired immune responses against HIV can be critically influenced by the timing and interval between prime and boost immunizations (IMs) (17). Primary Ag challenge has been shown to lead to the activation of naïve B cells, but more importantly to the generation of a memory B cell (mBC) pool that is vital for the secretion of class switched and high-affinity antibodies following Ag rechallenge (18). Upon reexposure to Ag mBC can rapidly differentiate into antibody secreting plasmablasts and plasma cells and thereby promote protective and long-lasting immune responses (19, 20). The timing of sequential IMs is thought to modulate the interplay between T and B cells influencing the quality and durability of induced humoral responses (21). The development of HIV antibodies with a high degree of somatic hypermutation requires Ag-specific B cells to repeatedly circulate between the light and dark zone of the germinal center (GC), a process driven by cognate CD4 T helper follicular (Tfh) cells and induction of appropriate cytokines and growth factors (20, 22, 23). The mechanisms underlying CD4 Tfh cell memory responses are poorly defined, and while expression of cognate Ag on B cells is thought essential at the peak of effector generation (24), longer term persistence beyond the expansion phase can lead to reduced memory functionality (25). Thus, effective generation of CD4 Tfh memory is likely Ag dose and time dependent, where revaccination before full contraction of the effector response may negatively impact on the boosting response. Therefore, understanding the time dependent impact of vaccination on the quality and durability of induced B and CD4 T cell responses may prove essential for the rational design of optimal homologous and/or heterologous prime-boost regimens against HIV.

To facilitate the rapid evaluation of prospective HIV vaccine regimens, small scale phase I clinical trials can be utilized to qualitatively and quantitatively measure vaccine-mediated humoral and T cell immune responses (17). The current homologous prime-boost study was designed to dissect the effect of a standard (6 months) versus delayed (12 month) boost on the quality of induced antibody response to a candidate HIV-1 envelope protein CN54gp140, coadministered with the TLR4-agonist glucopyranosyl lipid A-aqueous formulation (GLA-AF).

## Materials and Methods

### Ethical and Regulatory Approvals

The trial proposal, the trial-specific information provided to volunteers, the consent form and substantial protocol amendments (if applicable) were reviewed by a recognized Research Ethics Committee and by the Medicines and Healthcare products Regulatory Authority.

### Conduct of the Study

The study was conducted in compliance with UK Clinical Trial Regulations and any amendments, which include compliance with the principles of good clinical practice, and the study abided by the principles of the Declaration of Helsinki.

### Volunteer Information and Consent

All volunteers provided written informed consent to participate in the trial on the basis of appropriate information and with adequate time to consider the information and discuss the trial with the principal investigator or her delegate. Participants were asked to explain what the trial involved in their own words, to ensure the volunteer understood the intensity of the schedule and the issues associated with taking part in a trial of a candidate HIV vaccine. All volunteers were made aware that they were free to withdraw without obligation at any time and that such an action would not adversely affect any aspect of their medical care or legal rights.

### Study Vaccines

A recombinant uncleaved clade C HIV-1 envelope gp140 protein (CN54gp140) produced by Polymun Scientific (Klosterneuburg, Austria) to GMP specification, which has previously been reported to be immunogenic in a number of preclinical and clinical studies (26, 27), was used for the X001 clinical trial. The vaccine Ag CN54gp140 was administered intramuscularly into the deltoid muscle of the upper arm at a dosage of 100 µg CN54gp140 formulated with 5 µg GLA-AF [Infectious Disease Research Institute, Seattle, WA, USA (28)] in a total volume of 0.4 ml. GLA has been shown to enhance antibody responses in mice, non-human primates, and humans without causing adverse reactions (29, 30). Good adjuvanticity was observed in a previous influenza clinical trial where they used 2.5 µg GLA and a clinical trial of a leishmaniasis vaccine using 2 or 5 µg GLA in an oil-in-water emulsion formulation (GLA-SE), which was safe and well tolerated (30, 31). Since much of the reactogenicity of GLA-SE is believed to be associated with its oil-in-water form, the aqueous formulation GLA-AF was assumed to be less reactogenic. Subsequent animal studies revealed that GLA-AF has broadly equivalent adjuvant activity to GLA-SE and based on these findings for the current trial a 5-µg dose of GLA was selected (32). Further, good tolerability of GLA-AF at a dose of 5 µg in combination with the CN54gp140 Ag has been demonstrated in previous clinical trials conducted by us (27, 33).

### Trial Participants and Randomization

The trial was open labeled without placebo control, and we sought to enroll 12 healthy volunteers, male or female between the ages 18 and 45 who did not report high-risk behavior for HIV infection. They were randomly assigned to two groups of six each within a screening period of 42 days prior to the first vaccination. Trial participants were required to follow a rigorous visit schedule and depending on the study group underwent up to 17 blood draws (Tables S6 and S7 in Supplementary Material).

### IM and Sampling

Study participants in each group received three injections (priming phase) followed by a booster IM at 6 months (Group A) or 12 months (Group B) of the study vaccine CN54gp140 formulated with GLA-AF. The study schema is detailed in Table 1. Blood samples and mucosal secretions (cervicovaginal, urethral, rectal, and seminal) were collected at designated time points (Tables S6 and S7 in Supplementary Material) for immunogenicity and vaccine safety analysis.

**Table 1:** **Vaccine dosage and study schema**.

Group	*N*	Vaccine dosage^a^	Vaccine given at months				
			0	1	2	6	12
A	6	CN54gp140 + GLA-AF	+	+	+	+	−
B	6	CN54gp140 + GLA-AF	+	+	+	−	+

*^a^Vaccines: recombinant uncleaved clade C HIV-1 envelope gp140 protein (CN54gp140) administered intramuscularly into the deltoid muscle of the upper arm at a dosage of 100 µg CN54gp140 formulated with 5 µg glucopyranosyl lipid A-aqueous formulation in a total volume of 0.4 ml*.

### Primary and Secondary Outcomes

The primary endpoint was to evaluate the safety and tolerability of two vaccine regimens consisting of three initial injections of CN54gp140 (priming phase) at a dosage of 100 µg formulated with 5 µg GLA-AF and a final booster injection at 6 months (Group A) or 12 months (Group B). The secondary outcome was to assess B and T cell-mediated and antibody binding responses in the peripheral blood.

### Safety Assessments and Monitoring

Collected data were identified only by a volunteer identification number. Safety and tolerability were addressed by examining the overall rates of solicited and unsolicited adverse events and serious adverse events that were potentially associated with vaccination and the number of volunteers who experienced these events. All clinical and routine laboratory work was included in the safety analysis (Tables S1 and S2 and Figures S1 and S2 in Supplementary Material).

### HIV Testing

Study staff performed HIV testing and any associated pre- or posttest counseling, according to national guidelines at time points defined in the Schedules of Procedures (Tables S6 and S7 in Supplementary Material).

### Mucosal Secretion Collection

Cervicovaginal secretions were collected from female volunteers using the INSTEAD™ Softcup, a commercially available, self-inserted menstrual cup made of polyethylene. There is a risk of dislodging an intrauterine device (IUD) when removing the Softcup and women who have had toxic shock syndrome (TSS) should not use internal sanitary protection products. For these reasons, women with IUDs or a history of TSS were excluded from the study.

Urethral secretions were collected from male volunteers using a 2-mm diameter polyvinyl alcohol (PVA) sponge. Sponges could be self-inserted following explanation or were inserted by the clinical trials team at the NIHR/Wellcome Trust Imperial Clinical Research Facility at Hammersmith Hospital.

Rectal secretions were collected from male and female volunteers using a pre-moisturized (with 50 µl PBS) sponge.

Semen samples were either donated while within the ICRF or brought into the facility following donation at home (within 3 h of ejaculation). Participants were instructed to donate semen following 24 h of sexual abstinence.

Mucosal secretion collection occurred at the time points indicated in the Schedules of Procedures (Tables S6 and S7 in Supplementary Material), except at visits when female volunteers were menstruating, in which case cervicovaginal secretions were not collected.

## Peptide Array Analysis of Env-Specific IgG Responses

Microarrays were designed to cover recently transmitted global HIV strains and currently used vaccines, therefore including gp140 sequences from eight recently transmitted virus isolates (subtypes A, C, B, CRF01_AE and CRF02_AG), plus additional peptide variants for previously identified hot spots of IgG recognition as well as CN54gp140 and MVA-CMDR encoded Env immunogens (Ahmed et al., manuscript in preparation).^1^ Each of the overlapping 15mer peptides on the array is present in triplicate.

Analysis of serum on the peptide microarrays was carried out according to the manufacturer’s instructions with minor modifications (www.jpt.com). After initial blocking (10 min with T20-blocking buffer, Thermo Fisher), slides were incubated with diluted serum samples (1:100 in T20) for 2 h at RT with gentle shaking. Slides were washed five times with TBS-T (0.5% Tween20), and bound antibodies were detected with a mouse antihuman-IgG Dylight649 (JPT) secondary antibody (1:5,000 in T20) for 1 h at RT. After repeated washing, slides were dried and subsequently scanned on a GenePix 4000A scanner at 650 and 532 nm. Samples of each vaccine (T0—baseline, T1—14-day post third IM, and T3—14-day post fourth IM) were processed simultaneously.

Analysis and mapping was performed using GenePix Pro 6.0 (Molecular Devices) and R (ZZ). IgG responses against individual peptides were considered positive if the corresponding triplicate fluorescence intensity value was above 2,500 after subtraction of the pre-vaccination value in at least 25% of all vaccines.

### Processing of Mucosal Samples

Prior to processing the mucosal samples, the extraction buffer (EB) was prepared by adding 1 ml of 100× protease inhibitor cocktail set (Calbiochem, 539131) and 10% NaN_3_ (Sigma-Aldrich, S2002-25G, UK) to 100 ml sterile PBS.

Prior to analysis, Softcups containing cervicovaginal secretions in 50 ml tubes were removed from −80°C, allowed to thaw on ice overnight, and centrifuged at 400 × *g* for 15 min at 4°C. An equal volume of EB was added to the secretion samples, mixed and aliquoted.

The 2-mm diameter PVA sponge used for the collection of urethral samples, and 500 µl EB was added to the top chamber of a Costar Spin-X tube (Corning) and centrifuged for 15 min at 13,000 rpm and 4°C. The eluate was collected and aliquoted.

Tubes containing the rectal swabs were removed from −80°C, thawed on wet ice, and placed into a 50 ml tube containing a sterile falcon filter unit (70 µm). To each tube, 500 µl EB was added before centrifugation at 400 × *g* for 10 min at 4°C. To remove debris, the eluate was transferred into the top chamber of a Spin-X tube, centrifuged at 13,000 rpm for min at 4°C, and aliquoted.

Seminal secretions were transferred into one or two sterile, plastic, 2 ml microcentrifuge tubes before centrifugation at 1,200 × *g*, for 10 min, at 4°C. The supernatant (seminal plasma) was transferred into 2–3 prelabeled, sterile, plastic, 2 ml microcentrifuge tubes. All mucosal sample aliquots were frozen at −80°C until analysis *via* ELISA.

### Peripheral Blood Mononuclear Cell (PBMC) Sample Preparation

Peripheral blood mononuclear cells were isolated using density gradient separation from heparinized whole blood, used fresh or frozen (within 8 h of blood collection) in a mixture of fetal bovine serum (Sigma-Aldrich, St. Louis, MO, USA) and DMSO at a 90:10 ratio using a Kryo 560-16 rate controlled freezer (Planer, Sunbury-On-Thames, UK). PBMCs were stored in vapor phase liquid nitrogen at the IAVI Human Immunology Laboratory or St Mary’s Campus, Imperial College London.

### HIV-Specific Binding Antibodies—ELISA Platform

An extensive ELISA platform was developed and employed to measure both total and Ag-specific antibody responses in human plasma/serum samples and mucosal secretions. The protocols of the various Ig ELISAs are detailed in the Supplementary Material (Table S4 in Supplementary Material). In brief, Ag-specific gp140-binding antibodies were measured using a standardized ELISA platform. In plasma/serum samples Ag-specific IgA, IgM, IgG (total), and IgG subclasses were measured, while both total and Ag-specific IgA/G antibody responses were measured in the mucosal compartment (cervicovaginal, urethral, seminal, and rectal). A total of 96-well high binding MaxiSorp plates (Nunc) were coated with 50 μl/well recombinant CN54gp140 (Polymun Scientific) at 2.5 µg/ml in PBS for overnight at 4°C. As reference material, we used standard Igs (Table S4 in Supplementary Material), which were captured with a combination of antihuman kappa and lambda light chain specific mouse antibodies. These capture antibodies were coated onto the plates overnight at 4°C, and coated plates were washed four times with PBS-T before blocking with the appropriate assay buffer (Table S4 in Supplementary Material). Following further washing, diluted sera or mucosal samples were added to the Ag-coated wells and titrations of Ig standards were added to the kappa/lambda capture antibody coated wells at 50 μl/well and incubated for 1 h at 37°C. Plates were washed four times prior to the addition of secondary antibody and incubated for 1 h at 37°C. Plates were washed four times and developed with 50 μl/well of KPL SureBlue TMB substrate (Insight Biotechnology, UK). The detection of Ag-specific IgA/G in mucosal samples and the detection of Ag-specific IgA/M/G2 required a streptavidin-(poly)-HRP amplification step prior to TMB development (Table S4 in Supplementary Material). The reaction was stopped after 5 min by adding 50 μl/well of 1 M H_2_SO_4_, and the absorbance read at 450 nm on a KC4 spectrophotometer.

### IFN-γ T Cell ELISpot Assay

Cellular immunogenicity was assessed by IFN-γ T cell ELISpot using frozen PBMC. Prior to use, ELISpot plates, pre-coated with anti-IFN-γ antibody (Mabtech), were washed with PBS and blocked with RPMI supplemented with 10% heat-inactivated fetal calf serum (R10) for a minimum of 2 h at 37°C. PBMCs were plated out at 2 × 10^5^ cells/well in 50 µl. For CN54gp140-specific responses, two pools of 15mer peptides with 11 amino acid overlap (P1 and P2) matched to the HIV-1 sequence vaccine insert were plated in quadruplicate wells. Peptides were initially dissolved and pooled in DMSO before dilution in R10 with final assay DMSO concentration of 0.45% v/v. Eight negative no-peptide control wells were cultured in R10 supplemented with 0.45% DMSO. Positive controls in duplicate wells were cultured with 10 µg/ml phytohemagglutinin (Sigma-Aldrich, Dorset, UK) and a pool of CMVpp65-peptides. Each individual HIV and CMV peptide was at a final assay concentration of 1.5 µg/ml. PBMCs were thawed and rested overnight in RPMI 20% HIFCS prior to ELISpot assay setup. On the day of assay setup, viable PBMCs were counted using a Beckman Coulter Vi-cell XR counter and plated at 2 × 10^5^ cells/well in 50 µl and incubated for 16–24 h at 37°C, in 5% CO_2_. Spots were visualized using biotinylated anti-IFN-γ (Mabtech) combined with ABC streptavidin-HRP (Vector labs), and the color was developed using AEC substrate (Sigma). The reaction was stopped after 4 min by rinsing with tap water.

Plates were air dried overnight and the spots counted using an AID ELISpot Reader and version 5.0 software (AID GmbH). After quality control of the data, the mean number of spot forming units (SFU) in no-peptide wells was subtracted from test wells, and the results were expressed as the median net SFU/10^6^ PBMC. Responses at least 38 SFU/10^6^ PBMCs above background and at least 4× background were scored as positive. These response cutoffs were derived from assessing the 97.5% percentile of the peptide pool responses in PBMC from 95 HIV seronegative individuals.

### Intracellular Cytokine Staining and Analysis

To characterize T follicular helper cell responses, study samples at 2 weeks after the third priming injection and after the respective booster injection along with matched baseline samples were interrogated for phenotype and cytokine secretion using cross-qualified polychromatic flow cytometry. Cryopreserved PBMCs were thawed and rested overnight and resuspended at 1 × 10^6^ in 200 µl RPMI medium supplemented with 10% FBS and co-incubated with HIV-peptide pools, 1 μg/ml staphylococcal enterotoxin B (Sigma-Aldrich, Poole, Dorset, UK) or mock stimuli, BD Golgistop (Becton Dickinson, San Jose, CA, USA) and Brefeldin A (Sigma-Aldrich, Poole Dorset, UK) for 6 h at 37°C. Cells were stained with 50 µl LIVE/DEAD fixable aqua viability dye L34957 (Invitrogen, Paisley, UK), anti-CD4-V605 (Biolegend, UK), anti-CD45RO-PerCP-Cy5.5, anti-CXCR5-A488, anti-CXCR3-PeCF594, anti-PD-1-BV421, and anti-CCR7-PE-Cy7 (Becton, Dickinson, Oxford, UK) and stained intracellularly with anti-CD154-PE and anti-IFN-γ-APC (Becton, Dickinson, Oxford, UK) (see Table S5 in Supplementary Material). A minimum of 30,000 viable CD3+ CD4+ lymphocyte events were acquired on a BD LSR Fortessa, which had been set up according to the Perfetto protocol (34, 35). Data were analyzed using FlowJo (Treestar, Ashland, OR, USA), PESTLE, and SPICE (courtesy of Mario Roederer, Vaccine Research Center, National Institutes of Allergy and Infectious Diseases) software. An intracellular cytokine secretion responder was considered positive if the background-subtracted values were twice those of the mock stimulus for each CD4-cytokine combination.

### mBC Stimulation

To assess the magnitude of mBC responses, we adapted a previously published protocol (36). In brief, frozen PBMC from baseline (D0), 28 days post-priming phase (D84) and 28 days post-booster injection (D196 or D364) were resuspended at 1 × 10^6^ PBMC/ml in stimulation media composed of R10, 5 ng/ml IL-2 (Roche, UK) and 0.5 µg/ml R848 (Vac-r848, Invivogen, UK), cultured for 4 days and washed with R10 prior to plating.

### B-Cell ELISpot

Sterile 96-well ELISpot plates (MSIPS4510 from Millipore) were pre-wet with 15 μl/well 70% EtOH for 1 min. After washing the plates five times with 200 μl/well sterile water, 100 μl/well of the CN54gp140 Ag (5 µg/ml) and 100 µl of the capture antibody MT91/145 (Mabtech, at 15 µg/ml) were added to the ELISpot plate and incubated overnight at 4°C. To remove excess Ag, plates were washed five times with sterile 200 μl/well PBS before blocking with 200 μl/well R10 for 1–2 h, at 4°C. Following the removal of R10, cells were added at 10,000/well for total IgG and 125,000/well for Ag-specific response in R10. Each condition was tested in triplicate, and plates were incubated for 6 h at 37°C, 5% CO_2_. For spot development, plates were first washed six times with 200 μl/well PBS and then 100 μl/well of detection antibody MT78/145 (Mabtech, 1:500) was added at 1 µg/ml in PBS/0.5% FCS and incubated overnight at 4°C. The next day plates were washed five times with 200 μl/well PBS and 100 μl/well streptavidin-HRP (Mabtech, 1:1,000), diluted in PBS/0.5% FCS, was added and incubated for 1 h at room temperature. Fifteen minutes before developing the plate, the AEC substrate (BD Biosciences) was prepared according to the manufacturer’s instructions. After washing the plates five times with 200 μl/well PBS, 100 μl/well of the AEC substrate solution was added and incubated for a maximum of 5–10 min before stopping the spot development with tap water. SFU were counted using an automated AID ELISpot reader (Autoimmun Diagnostika GmbH, Strassberg, Germany).

### Customized Multiplex Dimer Assay

A customized multivariate multiplex assay was developed using a panel of gp140 Ags (Clade C: CN54, 1086, Clade A: UG37, Clade D: UG21–NIH AIDS Reagents) coupled to magnetic fluorescent multiplex beads (Bio-Rad, AU) as described previously (37). Biotinylated dimeric Fc-gamma-receptors (FcγRIIa-H131, FcγRIIIa-V158) were produced as previously described (16).

Coupled microspheres were premixed in multiplex assay buffer (PBS + 0.1% BSA + 0.05% Tween), creating a working mixture of 12.5 microspheres/bead type/μl. Using a black, clear bottom 96-well plate 40 µl of the working microsphere mixture (1,000 beads of each type/well) was added to 40 µl of 100× diluted serum (diluted in PBS). The plate was covered and incubated overnight at 4°C on a plate shaker. The plate was washed three times with 200 µl of assay wash (PBS-1×, 0.1% BSA, 0.5% Triton-100X) using a Bio-Plex Pro plate wash station (Bio-Rad, AU). Ag-specific antibody binding to dimeric FcγRs was detected by adding FcγRs followed by streptavidin PE, at 1.0 µg/ml with 50 μl/well. After 2 h incubation at room temperature on a shaker, the plate was washed three times with 200 µl of assay wash, and microspheres were resuspended in 100 µl of sheath fluid.

A Bio-Plex reader (Bio-Plex MAGPIX, Bio-Plex Manager 5.0, Bio-Rad) was used to detect the microspheres, and binding of PE was measured to calculate a median fluorescence intensity (MFI). Background signal, defined as the average MFI observed for each microsphere set when incubated with the PE-conjugated detection reagent in the absence of clinical antibody sample, was subtracted from the MFI for each sample.

### HIV Neutralization

Sera were tested for neutralizing activity against HIV-1 at Monogram Biosciences (San Francisco, CA, USA) as described previously (38, 39). A panel of viruses (92BR020, 92TH021_043, 93IN905_040, 94UG103_023, MGRM-C-026_024, SF162, TH023) covering clades A, B, C, E, and including the neutralization sensitive laboratory strain NL43 and the primary isolate JRCSF, was used. Neutralizing activity is displayed as the percent inhibition of viral replication (luciferase activity) at each antibody dilution compared with the specificity control (aMLV). Titers were calculated as the reciprocal of the plasma dilution conferring 50% inhibition (IC50).

### Statistical Methods

Statistical analysis was carried out using Prism 7.0a (GraphPad, CA, USA) or the R software (R3.3.2) for statistical programming (40).

## Results

### Sequential Intramuscular Injections of the Clade C ENV Protein CN54gp140 Appear Safe in Healthy Adult Volunteers

**Figure 1:**
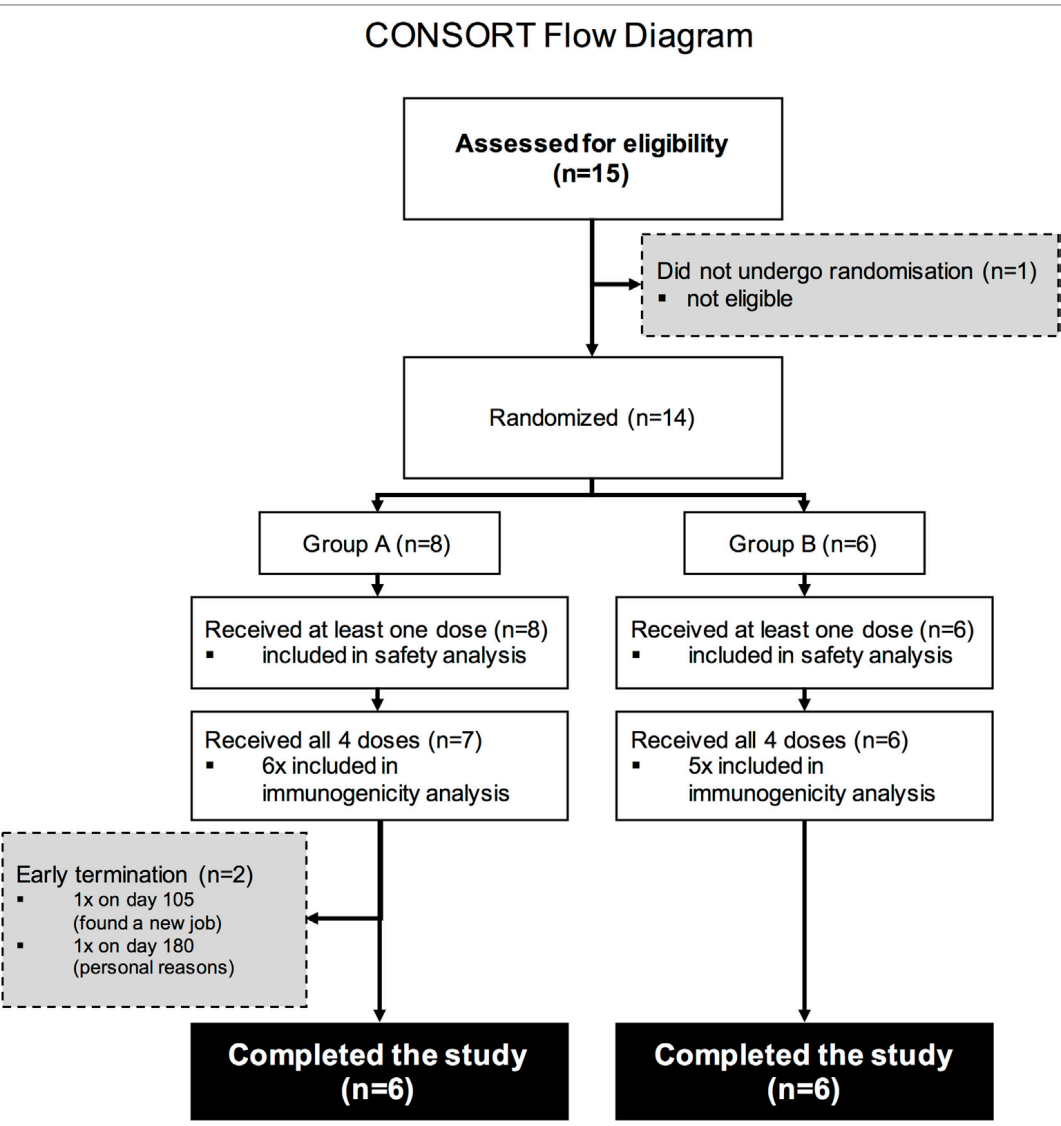
**CONSORT Flow diagram**. Numbers of participants recruited into the trial. Fifteen individuals were screened for the study, 14 were enrolled into the study (8 were enrolled into Group A, 6 into Group B), and 13 volunteers received all four intramuscular immunizations.

Fifteen study volunteers were assessed for eligibility with 14 deemed eligible according to the pre-defined inclusion and exclusion criteria. Two participants in study Group A withdrew due to personal reasons and were not included in the final immunogenicity analysis (Figure 1).

The majority of participants were male, and the mean age was 27.2 years (Table S1 in Supplementary Material). Overall, the vaccine was safe and well tolerated. Table S2 in Supplementary Material shows the proportion of volunteers with Grade 2 or higher reactogenicity events through day 7 post any vaccination. Most systemic reactogenicity events (fever, malaise, myalgia, headache, nausea, and vomiting) were graded as Grade 1 or Grade 2. The overall frequency of any Grade 2 or higher systemic reactogenicity following any vaccination was 42.9% (95% CI: 17.7–71.1; Table S2 in Supplementary Material). One Group B volunteer, X00143007, reported Grade 3 malaise on Day 2 after the third vaccination. All other reactions were Grade 1 or Grade 2 among volunteers in both study groups. All local reactogenicity (pain, tenderness, erythema/skin discoloration, and swelling/hardening or thickening) were graded as present or Grade 1.

### Systemic Vaccine-Induced Humoral Response

**Figure 2:**
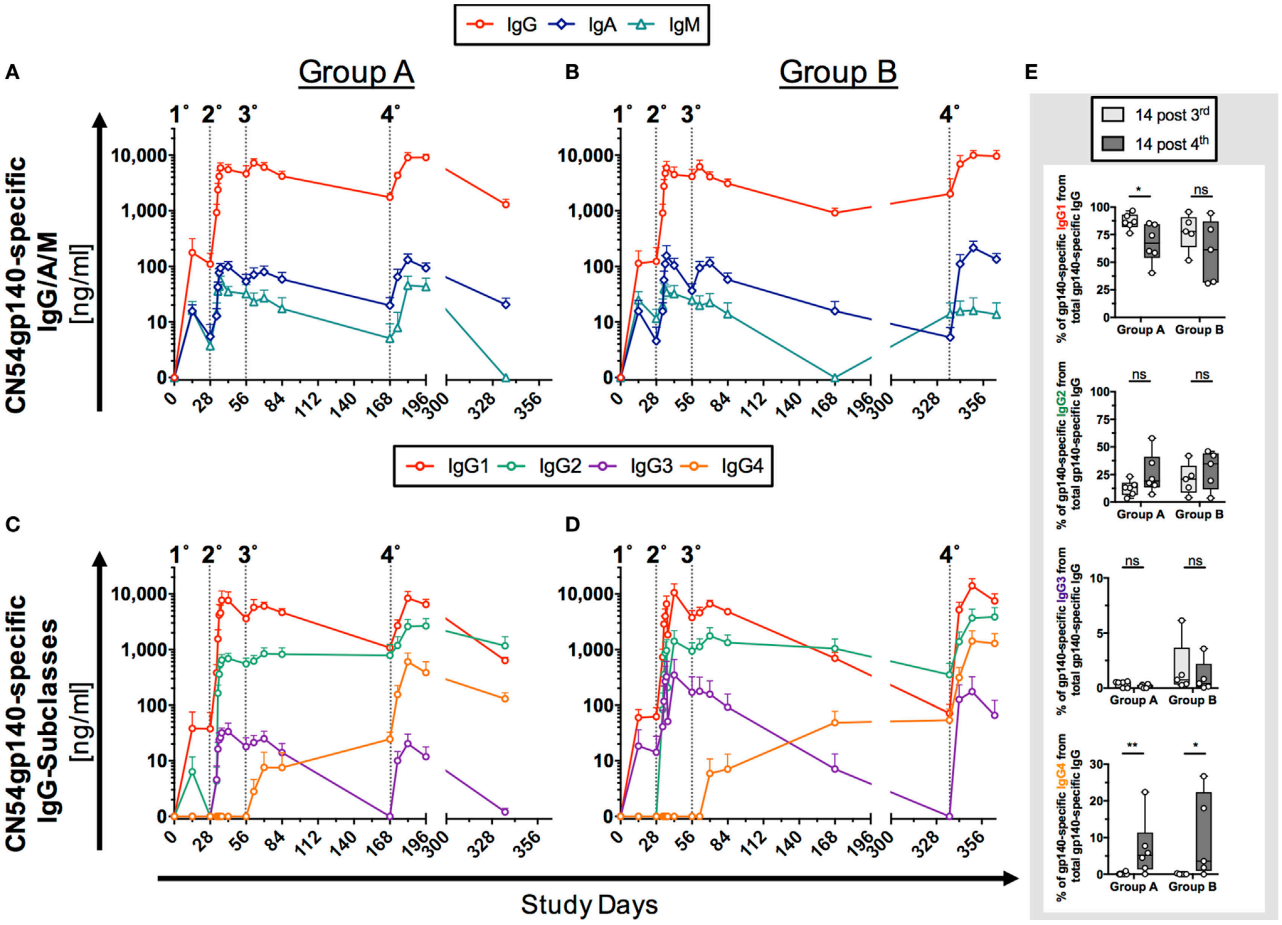
**Antigen (Ag)-specific humoral responses in human serum samples following homologous prime-boost vaccination regimens**. Participants received 3× priming immunizations (IMs) of CN54gp140 adjuvanted with GLA-AF on days 0, 28, and 56, followed by a booster injection at day 168 (Group A) or day 336 (Group B). Indicated by dotted line and numbers 1°–4°. **(A,B)** CN54gp140-specific IgG, IgA, IgM and **(C,D)** IgG-subclass concentrations were measured in human serum/plasma and expressed as mean ± SEM. **(E)** The sum of the four IgG subclasses was calculated and used to determine the percentage of CN54gp140-specific IgG-subclass antibodies 14 days after the priming phase (D70) and 14 days after the respective booster injections (D182/D350) for both Groups A and B. The proportion of Ag-specific IgG1 was decreased, while Ag-specific IgG4 levels were significantly increased after the respective booster IMs. Non-parametric Mann–Whitney test (*p < 0.05, **p < 0.01).

The humoral response to two homologous prime-boost HIV-vaccine regimens (Group A, *n* = 6; Group B, *n* = 5) was initially assessed by Ag-specific ELISA before and after sequential intramuscular IMs with the clade C ENV protein CN54gp140 (Figure 2). A low responder in Group B (Figure S3 in Supplementary Material) with significantly reduced levels of Ag-specific antibody levels was excluded as an outlier from the immunogenicity analysis in Figure 2.

The initial priming phase (first three IMs, Table 1) was common to both groups. Ag-specific IgG, IgA, and IgM antibodies were detected as early as 14 days after the first priming IM in both groups. IgG responses were significantly higher than IgA and IgM responses (*p* < 0.0001, Group A and B). Peak Ag-specific IgG, IgA, and IgM levels were reached after second IM, while the third IM had little additional impact on the magnitude of the response (Figures 2A,B). The decay in Ag-specific levels (IgG and IgA) between days 84 and 168 was similar for both groups (Group A/B: 2.4-fold/3.4-fold for IgG and 3-fold/3.7-fold for IgA). Although IgM levels appeared lower at day 168 in group B relative to group A, the differences were not statistically significant (*p* > 0.9999), where levels for both groups were at the lower limits of detection. In the boosting phase the fourth IM at 6 months (Group A) and 12 months (Group B) had a similar impact on the magnitude of boosted responses with no statistical differences between the respective endpoints of Ag-specific IgG, IgA, and IgM (Group A versus Group B: IgG: *p* > 0.99, IgA: *p* > 0.99, IgM: *p* = 0.56).

Further subclass analysis was performed to dissect IgG-subclass responses. Ag-specific IgG1/2/3 were detected as early as 14 days after the first IM on D0 (Figures 2C,D). Ag-specific IgG1/2 antibodies dominated the antibody profile in both groups and were detectable across all visits 14 days post the first IM. Interestingly, while the third IM had no apparent impact on Ag-specific IgG1/2 levels, it induced Ag-specific IgG4 levels in both groups, and these continued to rise between days 84 and 168 (Figures 2C,D) and were significantly boosted by the respective fourth IM (Figure S4 in Supplementary Material). In contrast, concentrations of Ag-specific IgG3 rapidly declined after the third IM to below the detection limit of the assay but were re-boosted to similar levels following the fourth IM (D168, Group A and D336, Group B). Intriguingly Ag-specific IgG2 levels appeared to be better sustained over days 84–168 in both groups than IgG1, which decayed 4.3-fold (Group A)/6.9-fold (Group B) and was of similar magnitude to IgG2 by day 168 in Group A (Figure 2C; IgG1/IgG2 ratio = 1.4), dropping below the levels of Ag-specific IgG2 by day 336 in group B (Figure 2D; IgG1/IgG2 ratio = 0.2). There was no apparent difference in Ag-specific IgG-subclass response to early (6 months, Group A) and late (12 months, Group B) boosting IMs (fourth IM); however, these re-boosted levels of Ag-specific IgG in both groups to levels observed after the second IM.

### Mucosal Ag-Specific Antibody Levels Directly Reflect Systemic Responses

**Figure 3:**
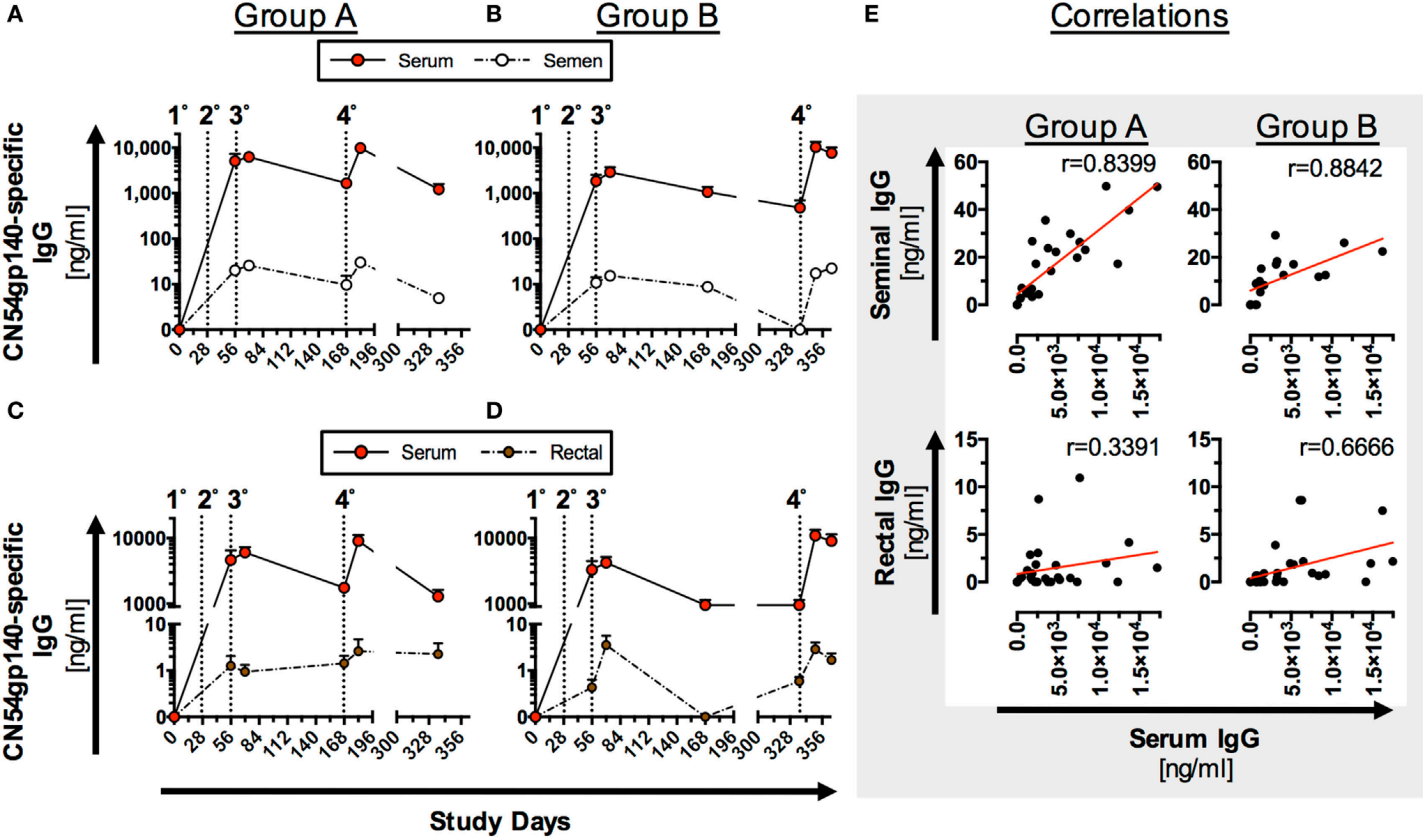
**Antigen (Ag)-specific humoral responses in seminal and rectal secretions following homologous prime-boost immunization (IM)**. Participants received 3× priming IMs of CN54gp140 adjuvanted with GLA-AF on days 0, 28, and 56, followed by a booster injection at day 168 (Group A) or day 336 (Group B); indicated by dotted line and numbers 1°–4°. **(A–D)** CN54gp140-specific IgG was measured in human serum/plasma, seminal (*n* = 8) and rectal secretions (*n* = 11) and expressed as mean ± SEM. **(E)** Ag-specific IgG levels in seminal secretions correlate significantly with systemic IgG in both groups, while correlation coefficients (*r*-values) were lower when comparing Ag-specific IgG in rectal and systemic compartments. Spearman’s rank correlation coefficient (*p* < 0.0001).

Antigen-specific mucosal antibody levels were determined using optimized sampling protocols and a high sensitivity ELISA assay. Reference values for total IgG and IgA for the different mucosal secretions are given in Figure S5 in Supplementary Material. Low levels of Ag-specific IgG antibodies could be detected in seminal and rectal secretions (Figure 3) and, for the four female participants, in cervicovaginal secretions (Figure S6 in Supplementary Material).

Mucosal antibody levels were significantly lower than Ag-specific IgG in serum samples (Figures 3A–D), but significantly correlated (*p* < 0.0001) with Ag-specific IgG in serum samples from both groups (Figure 3E). Response curves in mucosal secretions and the systemic compartments were identical, with a relatively constant differential between mucosal and systemic antibody levels between the respective compartments in both study groups (Figures 3A–D). Comparable trends were observed for both Groups A and B (Figures 3A–D) with no statistical difference in peak Ag-specific mucosal antibody levels 2 weeks post the fourth IM. Ag-specific IgG was below the limit of detection in urethral samples and reflects the low level of detected total IgG (Figure S5 in Supplementary Material) by this method. None of the mucosal samples exhibited detectable levels of Ag-specific IgA (data not shown), reflective of the low serum IgA responses (Figure 2).

### Vaccine-Induced Antibodies Target the V3 Region of the Clade C CN54gp140 ENV Protein

**Figure 4:**
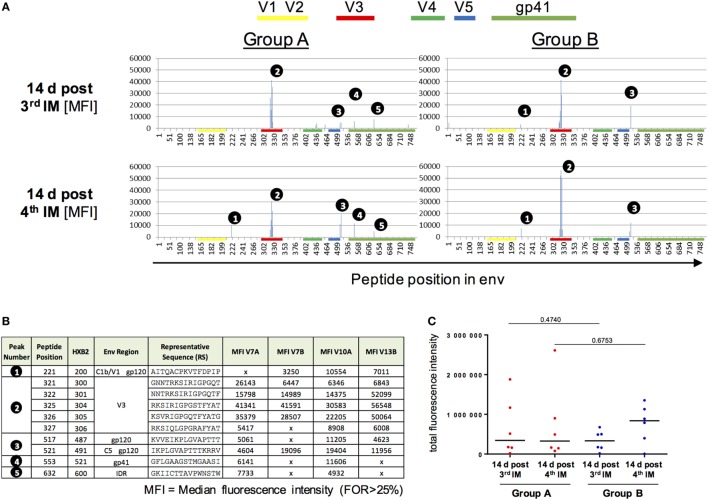
**Peptide array analysis of Env-specific IgG responses**. **(A,B)** Specificity of serum IgG responses at 14 days after the third and fourth intramuscular immunization (IM) for Group A and B. Shown is the median fluorescence intensity (MFI). IgG responses against individual antigenic regions were considered positive if the corresponding fluorescence intensity was above 2,500 after subtraction of the prevaccination value (D0). The MFI was then calculated for region-specific IgG responses occurring in at least 25% of vaccines. **(C)** Comparison of total fluorescence intensity of ENV-specific serum antibodies 4 days post third/fourth IM for Group A and B showed no significant differences (Mann–Whitney test).

Vaccine-induced IgG responses toward linear epitopes in the HIV Env were mapped using a customized peptide microarray consisting of overlapping linear 15mer Env peptides 2 weeks after third and fourth IM in relation to baseline (Figure 4).

Both groups, irrespective of the interval between booster injections, showed similar IgG Env-peptide recognition patterns (Figure 4A) both before and after the fourth IM. No difference in epitope recognition between the two groups was observed. A dominant recognition of the V3 tip region (peak number 2) by all vaccines can be observed after the three priming IMs. The longer boosting interval (Group B) was associated with an apparently higher median response to the V3 tip when compared to Group A. This difference, however, was not significant due to the high inter-individual variation in the IgG response toward the V3 tip (Figure S7 in Supplementary Material). IgG responses toward other regions within gp120 (peaks number 1 and 3, Figure 4B) were of lower magnitude and observed with varying frequency (33–83% of all vaccines) at both time points and in both groups. Recognition of gp41 only seems to occur in Group A, albeit already after three IMs and therefore seems to be due to interindividual differences in the IgG response rather than attributable to the boosting interval. There were no significant differences between the two groups in the total sum of fluorescence intensity values for all peptide variants included in the array (Figure 4C).

### Comparison of Fc-Receptor Binding Characteristics of CN54gp140-Vaccine-Induced Serum Antibodies between Study Groups

**Figure 5:**
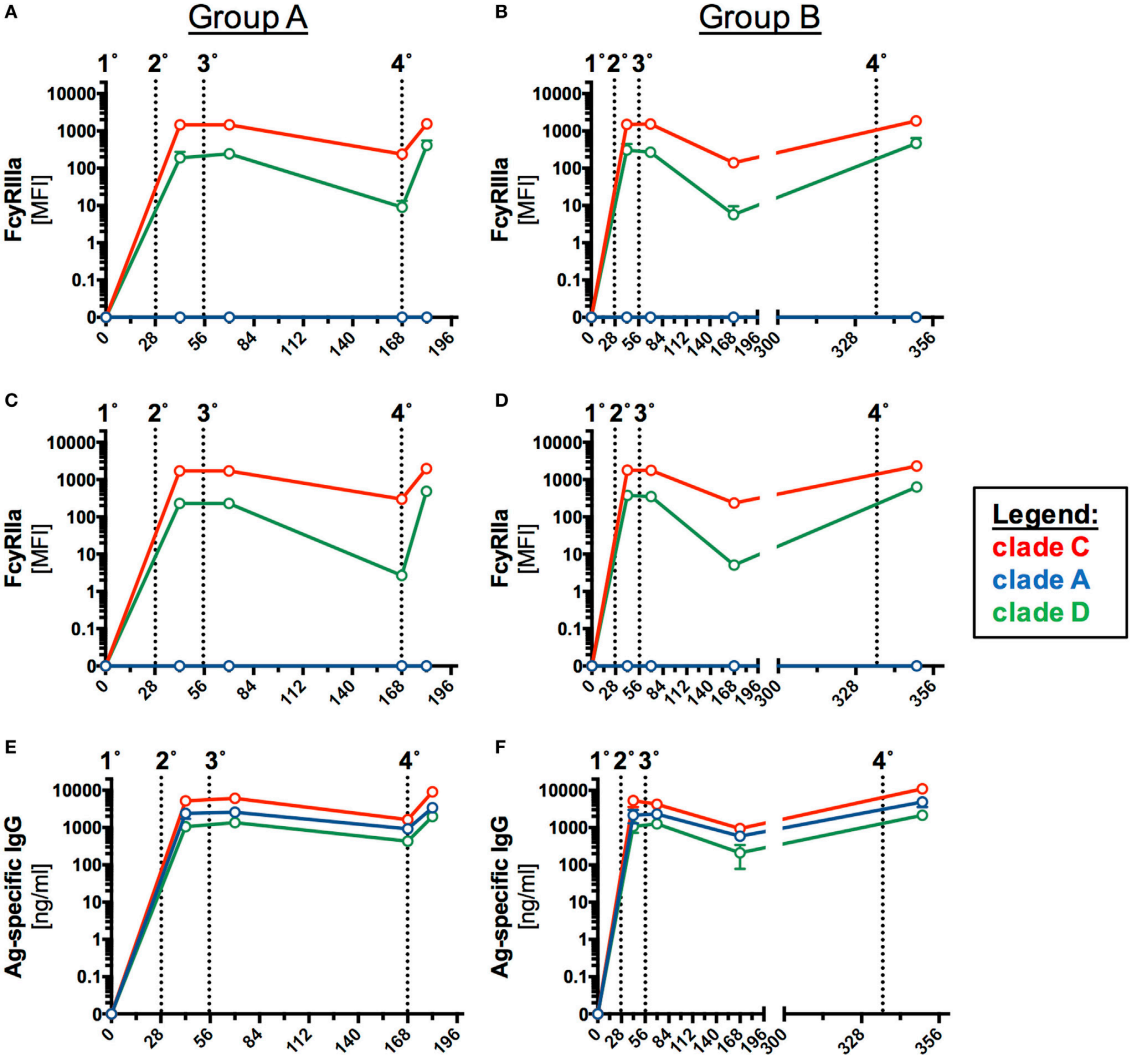
**FcγR-binding profiles and breadth of antigen (Ag)-specific humoral responses in serum samples following homologous prime-boost immunization**. The ability of vaccine-induced antibodies recognizing clade C.CN54gp140, A.UG37gp140, and D.UG21gp140 human immunodeficiency virus (HIV) proteins to bind dimeric **(A,B)** FcγRIIIa or **(C,D)** FcγRIIa was assessed in a customized multiplex dimer assay. **(E,F)** For further comparison, HIV-binding antibodies **(E,F)** were determined for the three selected clades for both study groups (A, *n* = 6 and B, *n* = 5).

Even though vaccine-elicited antibodies did not neutralize virus in this study (Table S3 in Supplementary Material), we sought to further investigate the ability of Ag-specific antibodies to crosslink Fc receptors. Recently, the role of non-neutralizing HIV antibodies in vaccine-mediated protection has gained prominence as they can induce important Fc effector-mediated functions. We have previously developed an assay using recombinant dimeric Fc receptors that provide an indirect readout for influenza-specific ADCC and phagocytosis (15, 16). Here, we investigated any differences in the ability of vaccine-induced serum antibodies to interact with dimeric Fc receptors FcγRIIIa and/or FcγRIIa after binding to various HIV-1 clade env proteins (Figures S8–S10 in Supplementary Material). For C.CN54gp140, A.UG37gp140, or D.UG21 proteins, both FcγR-profiles (Figures 5A–D) and Ag-specific IgG levels were compared (Figures 5E,F).

In both groups, clade C and D binding vaccine-induced antibodies were detected by the dimeric FcγRIIIa and FcγRIIa, while vaccine-induced antibodies specific to clade A did not crosslink dimeric FcγRIIIa and FcγRIIa (Figures 5A–D).

**Figure 6:**
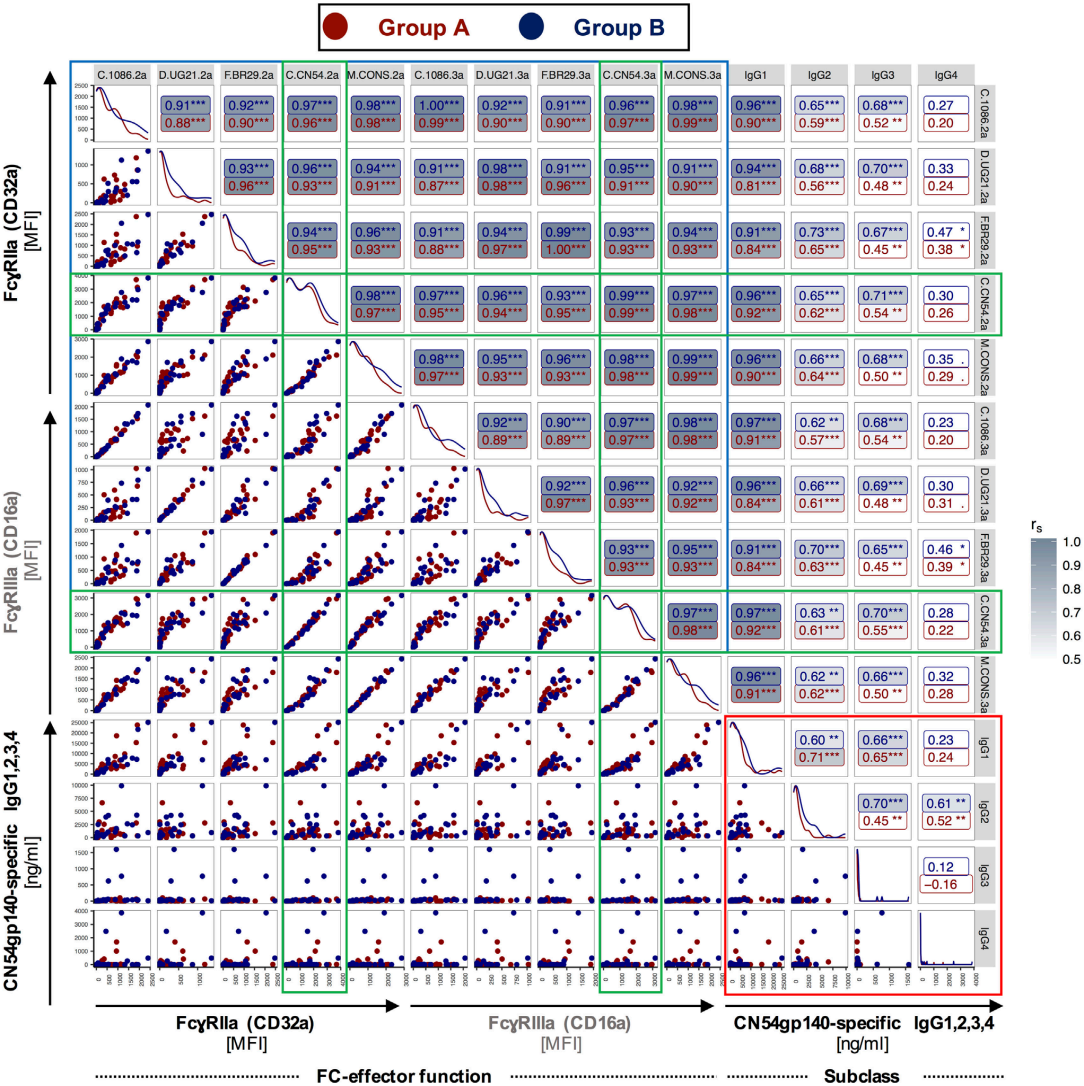
**Correlations between CN54gp140-specific IgG subclass measurements and FcγRIIa (CD32a)/FcγRIIIa (CD16a) binding profiles for Group A and B**. A customized multiplex assay was used to determine binding of dimerized FcγRIIa or FcγRIII following binding to different human immunodeficiency virus (HIV)-clade proteins in serum samples from Group A and B. For simplicity, the horizontal and vertical labels, indicating the various HIV-clade proteins, were labeled with the ending .2a or .3a, respectively, to designate FcγR dimers. Correlations between CN54gp140-specific IgG subclasses are boxed in red, and correlations between FcγRIIa/FcγRIIIa binding profiles are boxed in blue, while correlations directly related to the CN54gp140 antigen, which was used for serial immunizations, are boxed in green. C.CN54gp140-mediated engagement of both FcγRII- and FcγRIIIa-receptors was positively associated with CN54gp140-specific IgG1/2 in both groups. Both study groups exhibit identical isotypic and FcγR-binding profiles. *r*_S_—Spearman’s rank correlation coefficient (*p < 0.05, **p < 0.01, ***p < 0.001).

As expected, in an Ag-specific ELISA the largest proportion of vaccine-induced antibodies bound the clade C CN54gp140 immunogen (Figures 5E,F). Fourteen days after the final IM (Figures 5E,F, indicated by 4°), the concentration of vaccine-induced antibodies binding to clade A and/or clade D was about 3- or 4.5-fold lower, respectively, in Group A and 2- or 5-fold lower, respectively, in Group B (Figures 5E,F). Overall, the fingerprint of Fc-receptor binding, associated with different HIV-1 clades, was similar in both pattern and magnitude for the two study groups (Figure 5; Figure S7 in Supplementary Material) and FcR engagement following binding to five HIV-clade proteins correlated significantly with levels of CN54gp140-specific IgG1, 2 and 3 in both groups (Figure 6). Similarly to a previous study of HIV-controllers (41), the strongest positive correlation with nearly identical spearman coefficients between Group A and B was observed between FcγRIIIa/FcγRIIa dimerization and CN54gp140-specific IgG1 (Group A/B: ***p < 0.001, ***p < 0.001). The increases after the initial IM and subsequent declines observed in the Ab response profiles (Figures 5E,F) for the two groups are generally matched as features in the FcR activity profiles (Figures 5A–D). Interestingly, however, FcγR activity was not merely reflective of Ab titer since, the dimeric FcγRIIIa showed similar binding activity to opsonized clade C and clade D (Figures 5A,B), despite a profound reduction in antibody detecting clade D HIV Env (Figures 5E,F). Significantly, this suggests the antibodies that did cross-react against clade D Env, though low in titer, had high Fc-receptor binding ability.

### Both Prime-Boost Regimens Induced Ag-Specific mBC Responses in the Absence of Detectable T Cell Responses

**Figure 7:**
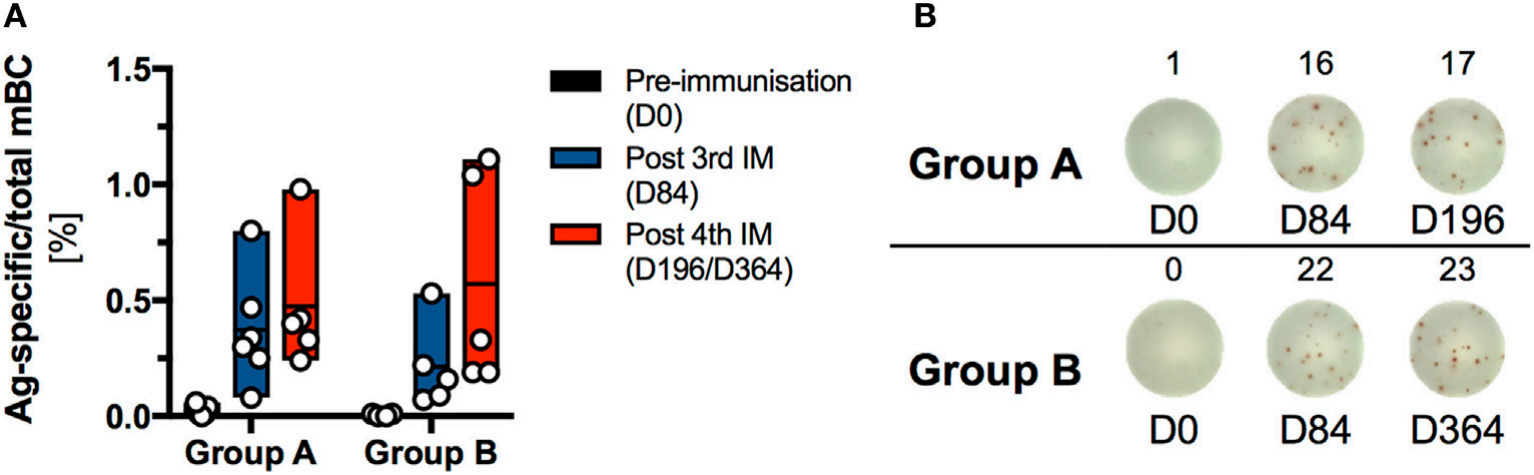
**Analysis of the memory B cell (mBC) response induced by sequential immunization with the human immunodeficiency virus-antigen CN54gp140**. Frozen peripheral blood mononuclear cells (PBMCs) were thawed and expanded using human IL-2 and R848 for 4 days. Expanded cells were assayed for antigen (Ag)-specific and total IgG-secreting ASCs by ELISPOT assay at 0, 84, and 196 or 364 days, respectively. Each sample was measured in triplicate, averaged and plotted as Ag-specific per total mBC. **(A)** Percentage of Ag-specific mBC per total IgG-secreting mBC. Due to the limitation in availability of PBMC at least one data point is missing for the following participants: S001 (D0), S002 (D0), and S013 (D0 and D196). **(B)** Ag-specific spot forming units/well (indicated by lower indices) Ag-specific mBC and total-IgG-secreting mBC for one selected donor from each group for days 0, 84, and 196 or 364, respectively.

An mBC ELISPOT assay was used to compare the dynamics and magnitude of Ag-specific mBC responses, generated by the two homologous prime-boost HIV-vaccine regimens (Group A, *n* = 6; Group B, *n* = 5) at 28 days after the third and fourth IM, respectively (Figure 7).

The frequency (mean ± SD) of Ag-specific mBC responses was similar for both vaccine regimens at 28 days after the third priming IM (Group A: 0.37 ± 0.25%; Group B: 0.21 ± 0.19%) and 28 days after the fourth IM (Group A: 0.47 ± 0.29%; Group B: 0.57 ± 0.46%). In each group, the highest responders exhibited an Ag-specific mBC frequency of about 1% total IgG-positive mBC. In contrast to the mBC response, no positive Ag-specific T cell responses were detected, *via* IFNγ-ELISpot or intracellular staining for CD154 and IFNγ, 14 days after the third and fourth IM in either group (Figure S11 in Supplementary Material).

## Discussion

The empirical process of optimizing IM schedules is likely key to the development of an efficacious HIV vaccine, where the interval between sequential IMs can prove critical in shaping the maturation of the induced adaptive immune response (42). This study was designed to analyze the effect of alternate booster regimens on antibody response profiles to CN54gp140 adjuvanted with GLA-AF. Study participants received a common three-dose intramuscular priming series followed by a final booster at either 6 or 12 months. Similar to previous studies (26, 27, 33), both regimens were observed to be safe and well tolerated, providing further evidence that CN54gp140-based vaccines exhibit a good safety profile.

The three initial doses of CN54gp140 were given at minimal intervals of 4 weeks and were anticipated to induce successive waves of Ag-specific antibody responses, where multiple administrations of protein vaccines are generally assumed to increase the magnitude of both T cell and antibody responses (43). In this study, peak IgG binding titers were reached following two priming IMs (at 0 and 28 days), while the administration of a third dose had no additional impact on the magnitude of Ag-specific IgG antibody titer. These data strongly suggest that two intramuscular priming doses are likely sufficient to induce strong antibody responses against CN54gp140. In this respect, previous studies using influenza hemagglutinin as an immunogen have indicated that the spacing between the first and second priming IM is similarly effective when administered at 14 rather than 28 days but is less efficient when this interval is reduced to 7 days (44). However, it has been suggested that a wider interval of 2 or 3 months between sequential IMs may be optimal to maximize the induction of a memory response (43). Interestingly, when using heamagglutinin as an Ag, the magnitude and breadth of the humoral response was increased by delaying the second priming dose to 6 months (44). Further studies are needed to identify the optimal timing of secondary administration of CN54gp140 to maximize the efficiency of two homologous priming injections. Furthermore, while the third priming IM had no apparent effect on the magnitude of induced binding antibody titers, we cannot exclude that this may have had an adverse impact on the durability of the induced antibody response. Indeed, previous studies using gp120 as an immunogen have suggested that repeated short-interval dosing reduced the durability of induced Ag-specific antibody levels (45). The possibility of dropping the third redundant priming IM has the potential to improve the durability of induced response warrants further investigation.

The common priming phase induced strong serological CN54gp140-specific IgG responses. These were mirrored by IgA and IgM responses in sera that were approximately two logs lower. IgG subclass analysis revealed a hierarchy in isotype responses where IgG1>IgG2>IgG3 with approximately a 1-log difference between each. The observed levels of IgG2 were higher than those reported for gp120 adjuvanted with alum in the Vax (003, 004) trials and in the RV144 trial (46), and likely reflect the adjuvant effects of GLA-AF inducing a Th1 biased profile (47). Importantly, using our optimized ELISAs, we were able to document IgG1–4 responses in all participants, whereas IgG2–3 responses were detected less frequently in the RV144 and VAX004 studies (46). Notably in our study, Ag-specific IgG4 only emerged after the third IM, in line with the known induction of IgG4 in response to repetitive Ag exposure and reflective of responses in VAX003 (46). Interestingly, despite having identical half-lives of 21 days (11), induced IgG2 levels appeared to have a slower decay rate to IgG1 between the priming phase and boosting in both groups. The mechanisms governing the different decay patterns could provide important insights into the mechanisms governing the longevity of induced humoral immunity.

A main focus of our study was to determine the impact of a long interval between the priming and boosting phase of vaccination. As previous studies using gp120 as an immunogen had indicated that boosting at 6 months was superior to that at 4 months (48), we elected to compare the response between booster IMs administered at 6 months (standard) or 12 months (late). We found no evidence to support that a long resting phase (12 months) had any impact on the magnitude of induced responses over the standard regime (6 months). IgG binding antibody levels post boosting were restored to similar levels to that observed after the priming phase but were not further increased. In contrast to our findings, a previous study using gp160 as an immunogen, adjuvanted with alum, and with a priming phase of 0, 1, and 6 months, reported that a delayed booster given at 18 months increased the magnitude of induced binding antibodies (49). However, responses were relatively low after the first three IMs in the latter study and likely reflect differences in the adjuvant potential of GLA-AF and alum.

We observed that for most antibody isotypes there was no apparent difference in Ag-specific antibody levels after the standard or late booster (total specific IgG, IgA, IgM, IgG1, and IgG3). However, Ag-specific IgG4 increased significantly in both groups after the final booster. Notably, this increase in IgG4 was not accompanied by a decline in IgG3 binding antibodies as reported for VAX003 (46). Analogous to previous HIV-vaccine studies our results link the late emergence of Ag-specific IgG4 antibodies with responses to repetitive boosting (46). This late emergence of IgG4 antibodies following repeated Ag exposure has been linked to the terminal position of the Cγ4 cassette (11, 50). Interestingly, we also observed an increase in IgG2 binding antibodies following boosting in both groups altering the relative IgG subtype proportions relative to those observed post third IM (Figure 2). The role of IgG2 in HIV immunity is unclear (9). Typically, IgG2 responses are more common against carbohydrate epitopes (11, 51) and may be better suited for the induction of anti-glycan responses associated with neutralization (although not observed here). Both IgG2 and IgA responses appear to be suppressed in HIV infection mediated by HIV nef, suggesting that this is a potential immune evasion mechanism (52). However, like IgG4, IgG2 is a poor activator of FcR engagement and thus poor at triggering ADCP and ADCC relative to IgG1 and IgG3 (46, 53, 54). It is unclear if the observed increase in IgG2 levels following the fourth IM would be further increased with additional boosters. In any event, the data provide another example that vaccine-induced immune responses are rarely static and have the potential to be modulated where additional boosters may be required to maintain protective antibody levels.

To further evaluate the quality of antibody responses, we used FcγRIIIa/FcγRIIa ectodomains to probe vaccine-induced serum IgG for its ability to engage Fc receptors critical to cellular functions such as ADCC or ADP (16). The importance of ADCC has been highlighted by the RV144 trial, in which robust HIV-specific ADCC responses were linked to the observed partial protection (7, 55, 56). In this study, we identified identical FcγR-binding profiles between the two study groups and found that both vaccine regimens induced comparable levels of Abs specific to both the clade C immunogen and a heterologous clade D Env protein capable of binding dimeric FcγRIIIa/FcγRIIa receptors. There was a relative robust ability of the cross-reactive antibodies to the clade D Env to bind both FcγRIIIa/FcγRIIa receptors despite very low binding antibody levels, suggesting these cross-reactive antibodies had a high capacity for binding Fcγ receptors. Cross-reactive binding Abs specific to clade A, however, were low and did not crosslink either Fc-receptor dimer. It is possible that the binding of vaccine-induced serum IgG to clade A was too transient for the detection of FcγR ectodomain engagement. Interestingly, the levels of FcγRIIIa/FcγRIIa receptor aggregation were similar post-boost in both groups, despite increasing levels of IgG2 and IgG4 (Figure 2). This contrasts to a previous analysis of VAX003 where boosting was associated with a drop in ADCC and ADCP function. The difference between the latter study and our own is that IgG3 binding antibody levels were similar post third and fourth IM, whereas in VAX003 IgG3 levels were decreased on boosting together with a concomitant rise in IgG4 level (46).

Complementary peptide array analysis revealed that most vaccine-induced IgG antibodies were specific to the V3 tip region with no significant differences between alternate boosting intervals. Despite the high prevalence of binding antibodies against CN54gp140 in both groups, we observed minimal neutralizing antibody responses against a panel of tier 1 HIV isolates, in line with our previous studies (26, 27, 33). Interestingly, both regimens also induced non-neutralizing binding antibodies against clade C and D and other envelope proteins. The demonstration of CN54gp140-mediated multi-clade specificity further expands the scope of CN54gp140-associated immune responses, as this was not examined in our previous clinical trials (26, 27, 33). The induction of strong antibody responses against the Env immunogen CN54gp140 is assumed to be dependent on Ag-specific T follicular helper cells. While both study regimens induced detectable Ag-specific mBC responses in the periphery, we did not detect systemic CD4 T-cell responses. This likely reflects that B cell help, provided by T follicular helper cells, is largely restricted to the GCs, whereas the frequency of circulating vaccine-induced Tfh cells is low and difficult to detect (57, 58). Recent studies have suggested that lymph node (LN) fine-needle aspirates may provide a more accurate assessment of vaccine-induced Tfh responses (59, 60). Direct probing of draining LNs could be especially useful to better understand how the circulation of GC B and T cells leads to the generation of specific and broader immune responses to gp140 immunogens.

In this study, mucosal samples were used to assess antibody levels in vaginal, genitourinary, and anorectal compartments. Encouraged by our previous findings (27), we developed more sensitive assays and subsequently identified a significant correlation between Ag-specific IgG response curves in the systemic and mucosal compartments. These highly similar profiles provide strong evidence that the frequency of seminal, rectal, and vaginal antibodies is reflective of the transudation of systemic antibodies to the mucosae. This passive transudation, occurred irrespective of the booster regimen, and supports the hypothesis that the induction of strong systemic responses *via* vaccination can elicit detectable antibody levels at mucosal surfaces. In this respect, mucosal antibody levels may provide an important surrogate of mucosal tissue levels, providing a key defense against HIV acquisition (61). The low magnitude of mucosal Ag-specific IgA directly reflects the low systemic levels of detectable Ag-specific IgA.

In summary, we applied innovative immunoassays to directly compare the effect of alternately timed booster regimens on the maturation of vaccine-induced humoral responses. The dissection of immuno-fingerprints of both booster regimens not only demonstrated that diverse Ag-specific HIV-1 humoral responses were effectively and safely induced but also revealed strikingly similar correlations between vaccine-induced IgG-subclass antibodies and Fc-receptor engagement between the two homologous prime-boost vaccine regimens. The significant similarities in response magnitude and antibody profiles, associated with both IM schedules, strongly support the feasibility of adopting the accelerated 6-month regimen for the rapid induction of immune responses against CN54gp140 with no apparent risk of diminishing response strength or Fc-receptor binding ability.

## Ethics Statement

The trial proposal, the trial-specific information provided to volunteers, the consent form and substantial protocol amendments (if applicable) were reviewed by a recognized Research Ethics Committee (REC) and by the Medicines and Healthcare products Regulatory Authority (MHRA). This study was carried out in accordance with the recommendations of UK Clinical Trial Regulations of the Medicines and Healthcare products Regulatory Authority (MHRA) and any amendments, which include compliance with the principles of Good Clinical Practice (GCP) with written informed consent from all subjects. All subjects gave written informed consent in accordance with the Declaration of Helsinki. The protocol was approved by the UK Clinical Trial Regulations committee of the Medicines and Healthcare products Regulatory Authority (MHRA).

## Author Contributions

RS, PM, and JG conceived the project. SK and PM designed and performed experiments, analyzed the data, and together with RS composed the manuscript. Experimental work was supported by LM and CP. YA and DK developed the ELISAs for measuring antibodies in mucosal secretions. JK, CB, and PH, with input from PM and SK, performed T cell assays. T cell data were analyzed by JK and LD. BW, PH, AC, and SK designed and performed a customized multiplex assay and preprocessed the data, which were analyzed by SK, AC, and DC. KH and CG provided the peptide array analysis. NS, TC, and SF managed the patient contact and sample collection from study participants. All the authors provided critical feedback on the manuscript prior to publication and have agreed to the final content.

## Conflict of Interest Statement

LD was employed by the EMMES Corporation (Rockville, MD, USA). All other authors declare no competing interests.

## Footnotes

^1^Ahmed et al. Dissecting the dynamics of the envelope-specific IgG response over 2 years of HIV infection. (Manuscript in preparation).
