# Supplementary material for: A Phase 1 Human Immunodeficiency Virus Vaccine Trial for Cross-Profiling the Kinetics of Serum and Mucosal Antibody Responses to CN54gp140 Modulated by Two Homologous Prime-Boost Vaccine Regimens
Source: Front Immunol. 2017 May 24;8:595. doi: 10.3389/fimmu.2017.00595 (PMC5442169; doi:10.3389/fimmu.2017.00595)
Supplement: Supplementary file 1 [file presentation_1.pptx]

## Slide 1
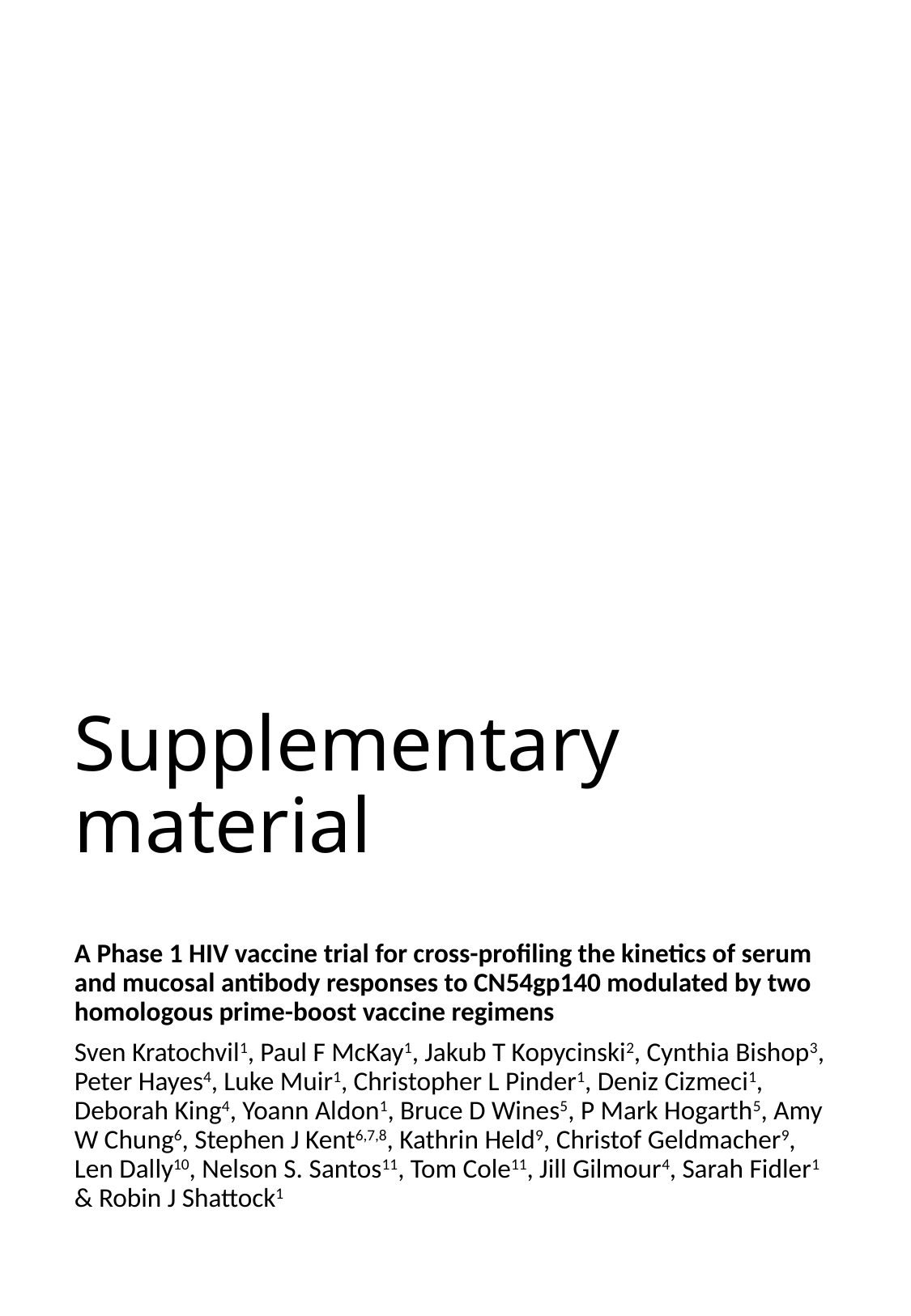

# Supplementary material
A Phase 1 HIV vaccine trial for cross-profiling the kinetics of serum and mucosal antibody responses to CN54gp140 modulated by two homologous prime-boost vaccine regimens
Sven Kratochvil1, Paul F McKay1, Jakub T Kopycinski2, Cynthia Bishop3, Peter Hayes4, Luke Muir1, Christopher L Pinder1, Deniz Cizmeci1, Deborah King4, Yoann Aldon1, Bruce D Wines5, P Mark Hogarth5, Amy W Chung6, Stephen J Kent6,7,8, Kathrin Held9, Christof Geldmacher9, Len Dally10, Nelson S. Santos11, Tom Cole11, Jill Gilmour4, Sarah Fidler1 & Robin J Shattock1

## Slide 2
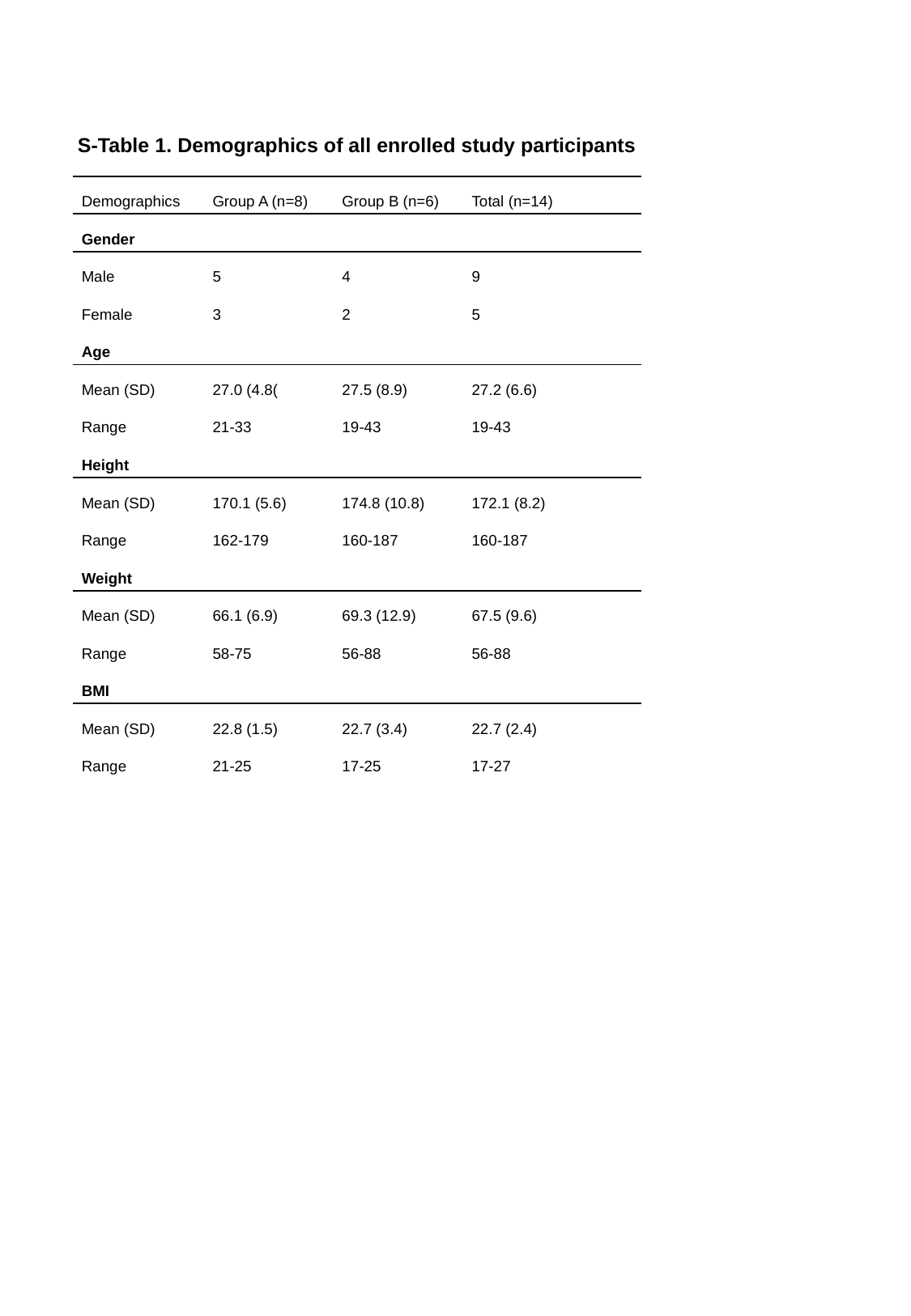

S-Table 1. Demographics of all enrolled study participants
| Demographics | Group A (n=8) | Group B (n=6) | Total (n=14) |
| --- | --- | --- | --- |
| Gender | | | |
| Male | 5 | 4 | 9 |
| Female | 3 | 2 | 5 |
| Age | | | |
| Mean (SD) | 27.0 (4.8( | 27.5 (8.9) | 27.2 (6.6) |
| Range | 21-33 | 19-43 | 19-43 |
| Height | | | |
| Mean (SD) | 170.1 (5.6) | 174.8 (10.8) | 172.1 (8.2) |
| Range | 162-179 | 160-187 | 160-187 |
| Weight | | | |
| Mean (SD) | 66.1 (6.9) | 69.3 (12.9) | 67.5 (9.6) |
| Range | 58-75 | 56-88 | 56-88 |
| BMI | | | |
| Mean (SD) | 22.8 (1.5) | 22.7 (3.4) | 22.7 (2.4) |
| Range | 21-25 | 17-25 | 17-27 |

## Slide 3
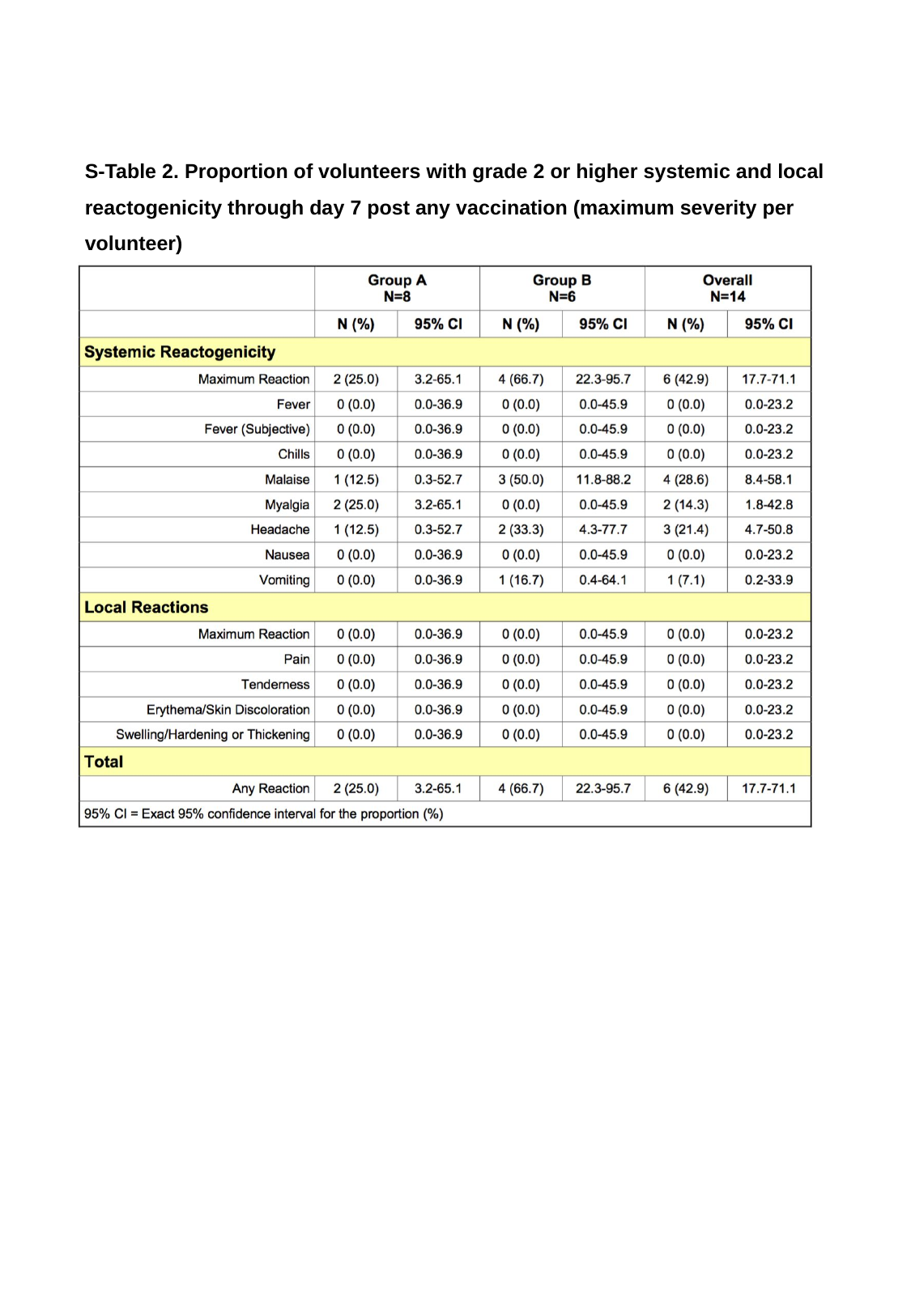

S-Table 2. Proportion of volunteers with grade 2 or higher systemic and local reactogenicity through day 7 post any vaccination (maximum severity per volunteer)

## Slide 4
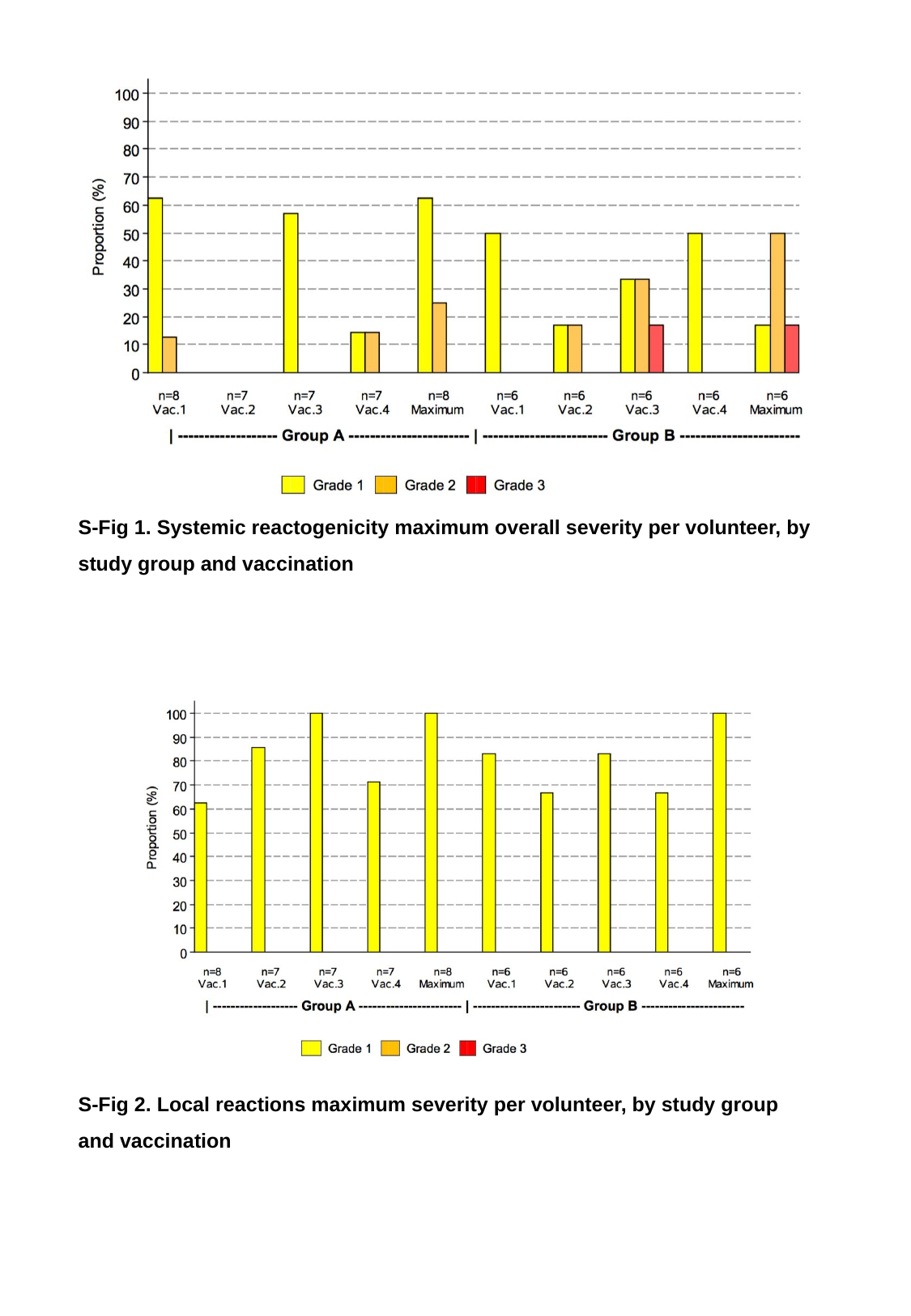

S-Fig 1. Systemic reactogenicity maximum overall severity per volunteer, by study group and vaccination
S-Fig 2. Local reactions maximum severity per volunteer, by study group and vaccination

## Slide 5
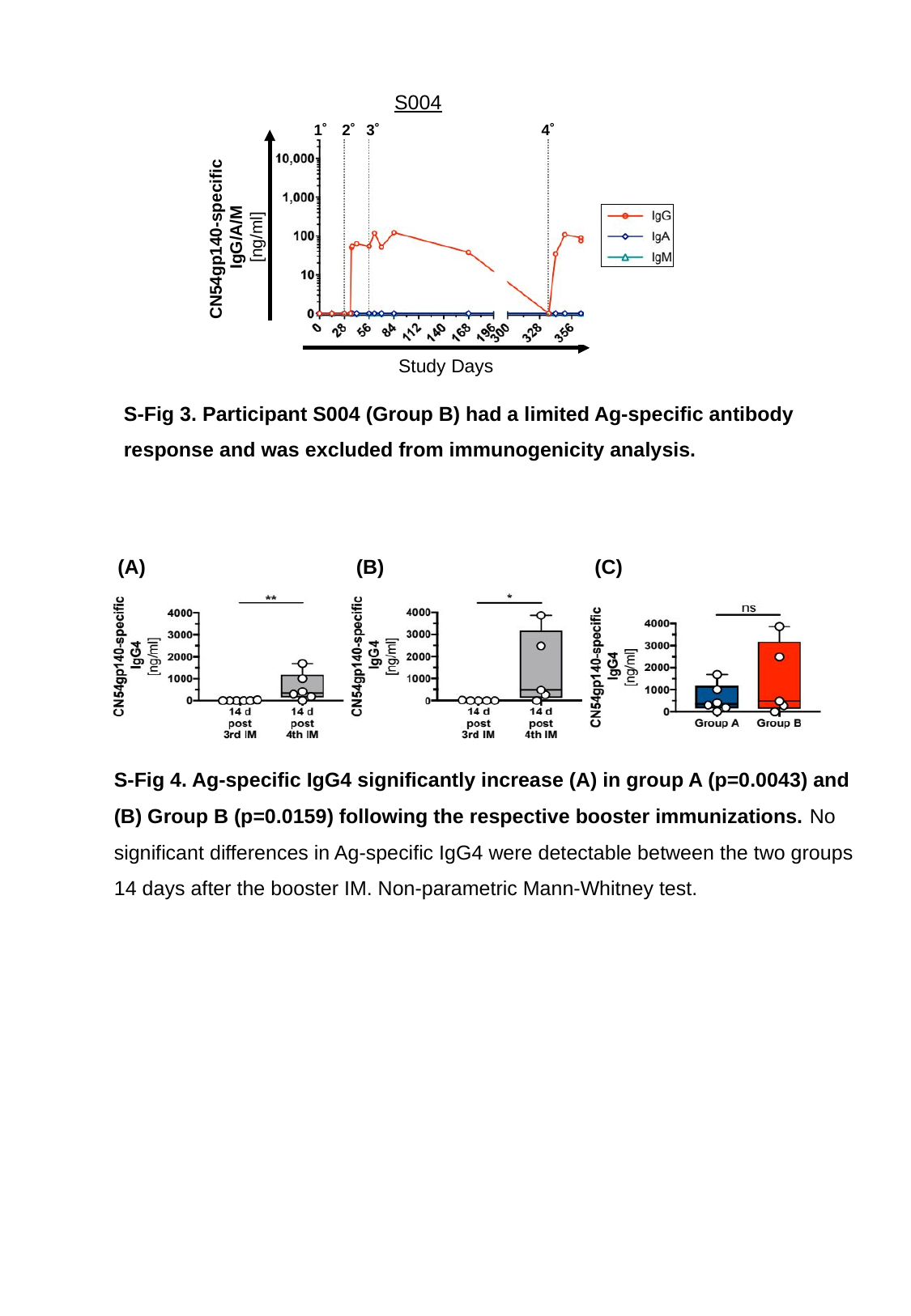

S004
1˚
2˚
3˚
4˚
CN54gp140-specific
IgG/A/M
[ng/ml]
Study Days
S-Fig 3. Participant S004 (Group B) had a limited Ag-specific antibody response and was excluded from immunogenicity analysis.
(A)
(B)
(C)
S-Fig 4. Ag-specific IgG4 significantly increase (A) in group A (p=0.0043) and (B) Group B (p=0.0159) following the respective booster immunizations. No significant differences in Ag-specific IgG4 were detectable between the two groups 14 days after the booster IM. Non-parametric Mann-Whitney test.

## Slide 6
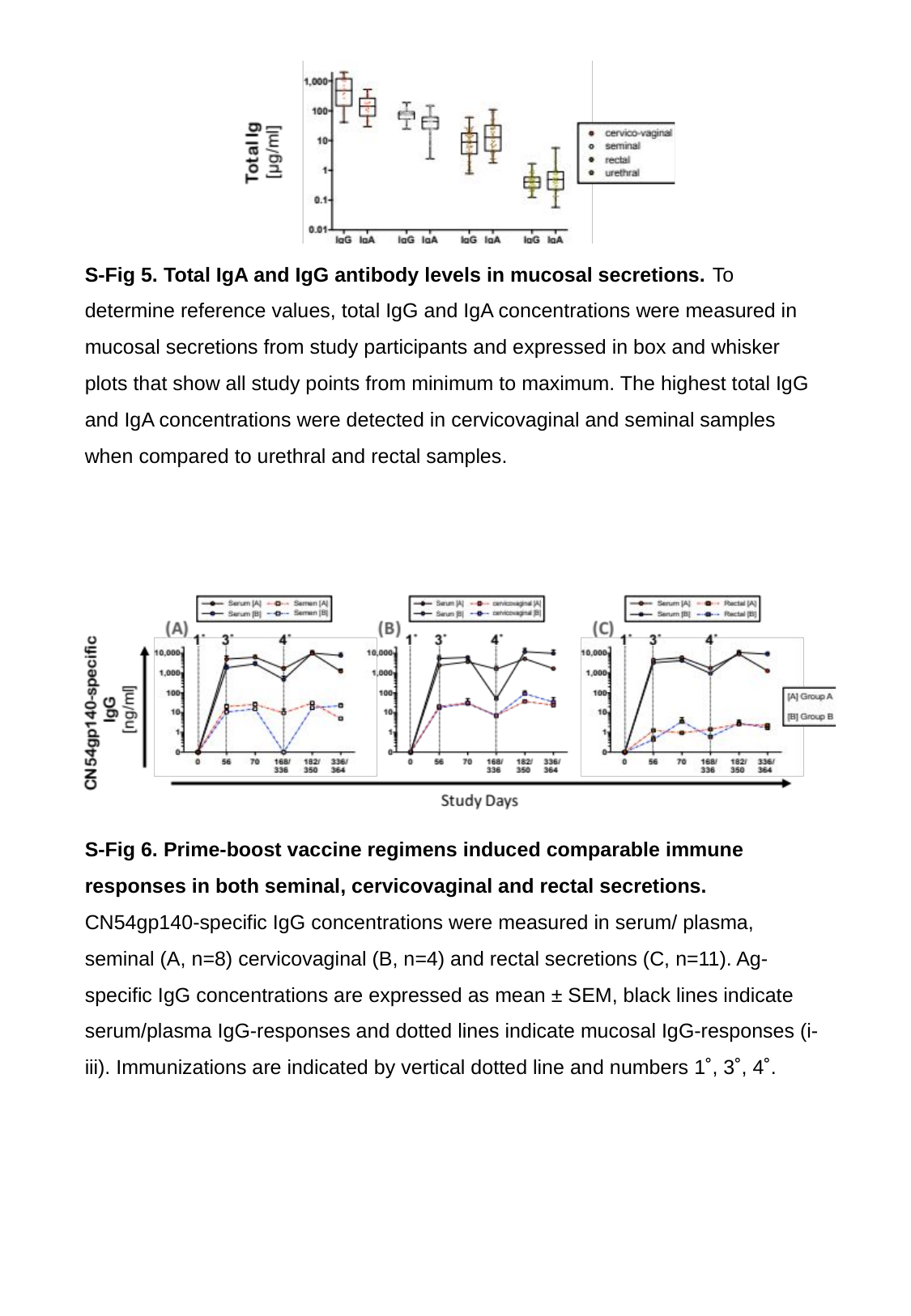

S-Fig 5. Total IgA and IgG antibody levels in mucosal secretions. To determine reference values, total IgG and IgA concentrations were measured in mucosal secretions from study participants and expressed in box and whisker plots that show all study points from minimum to maximum. The highest total IgG and IgA concentrations were detected in cervicovaginal and seminal samples when compared to urethral and rectal samples.
S-Fig 6. Prime-boost vaccine regimens induced comparable immune responses in both seminal, cervicovaginal and rectal secretions. CN54gp140-specific IgG concentrations were measured in serum/ plasma, seminal (A, n=8) cervicovaginal (B, n=4) and rectal secretions (C, n=11). Ag-specific IgG concentrations are expressed as mean ± SEM, black lines indicate serum/plasma IgG-responses and dotted lines indicate mucosal IgG-responses (i-iii). Immunizations are indicated by vertical dotted line and numbers 1˚, 3˚, 4˚.

## Slide 7
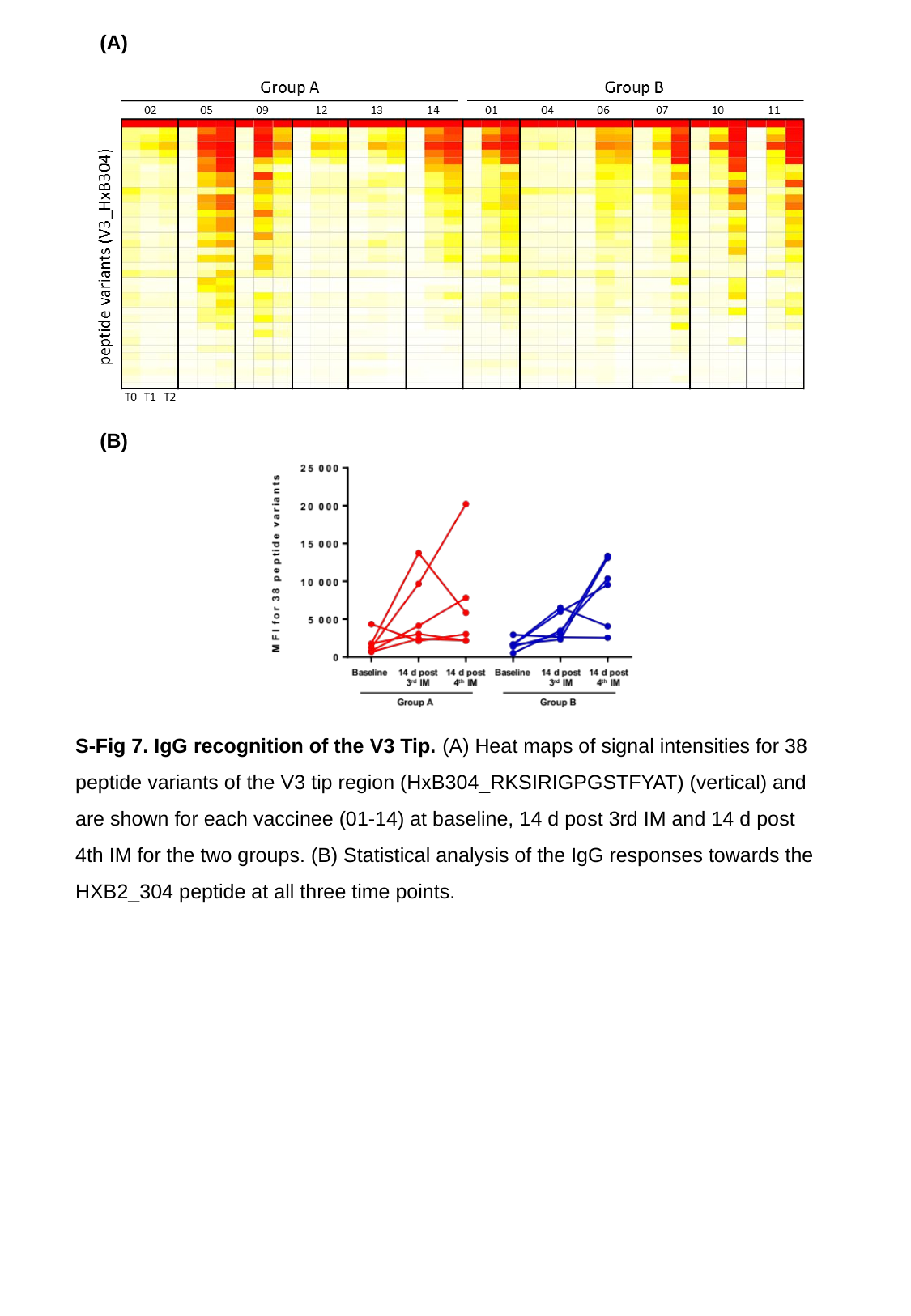

(A)
(B)
S-Fig 7. IgG recognition of the V3 Tip. (A) Heat maps of signal intensities for 38 peptide variants of the V3 tip region (HxB304_RKSIRIGPGSTFYAT) (vertical) and are shown for each vaccinee (01-14) at baseline, 14 d post 3rd IM and 14 d post 4th IM for the two groups. (B) Statistical analysis of the IgG responses towards the HXB2_304 peptide at all three time points.

## Slide 8
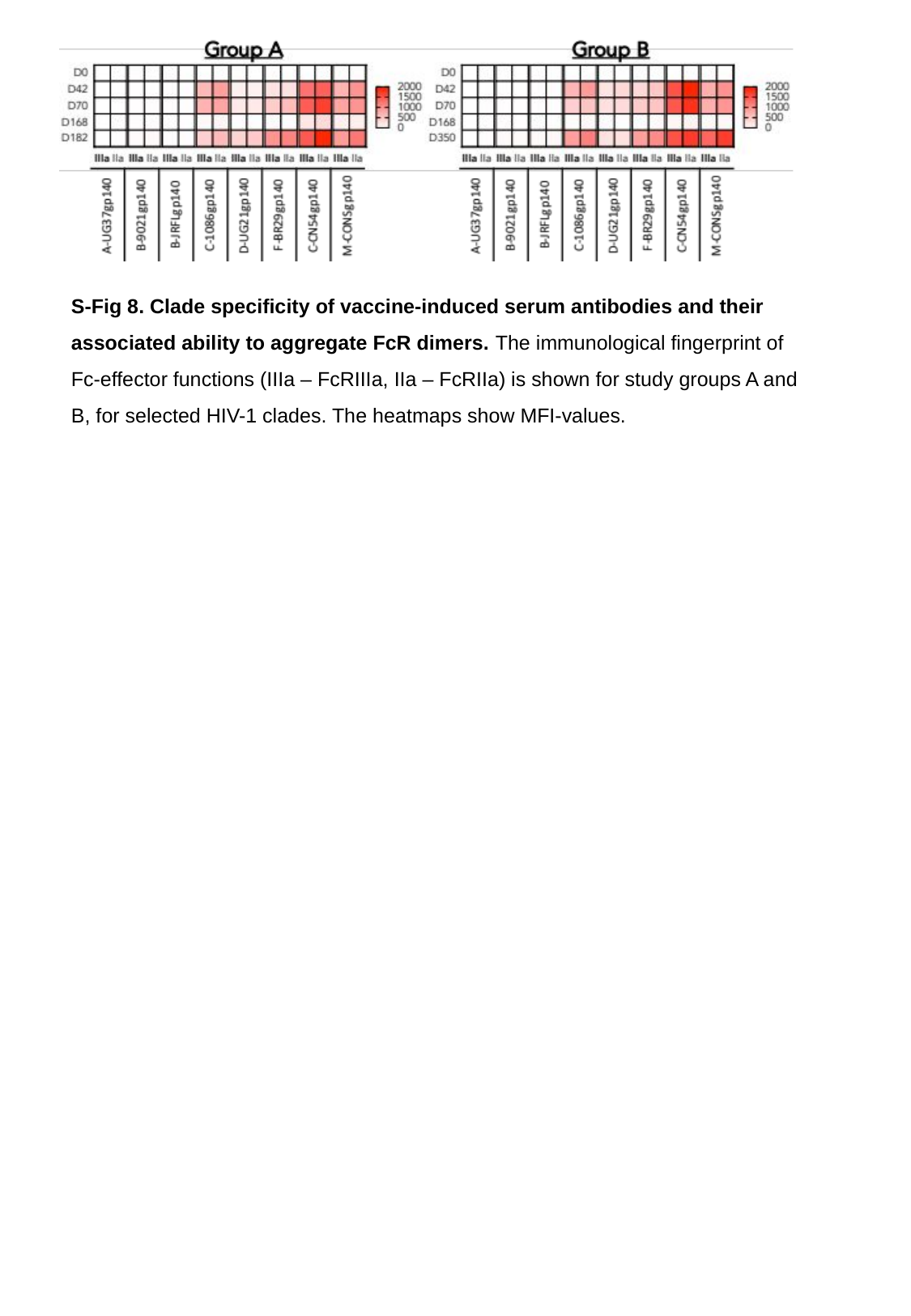

S-Fig 8. Clade specificity of vaccine-induced serum antibodies and their associated ability to aggregate FcR dimers. The immunological fingerprint of Fc-effector functions (IIIa – FcRIIIa, IIa – FcRIIa) is shown for study groups A and B, for selected HIV-1 clades. The heatmaps show MFI-values.

## Slide 9
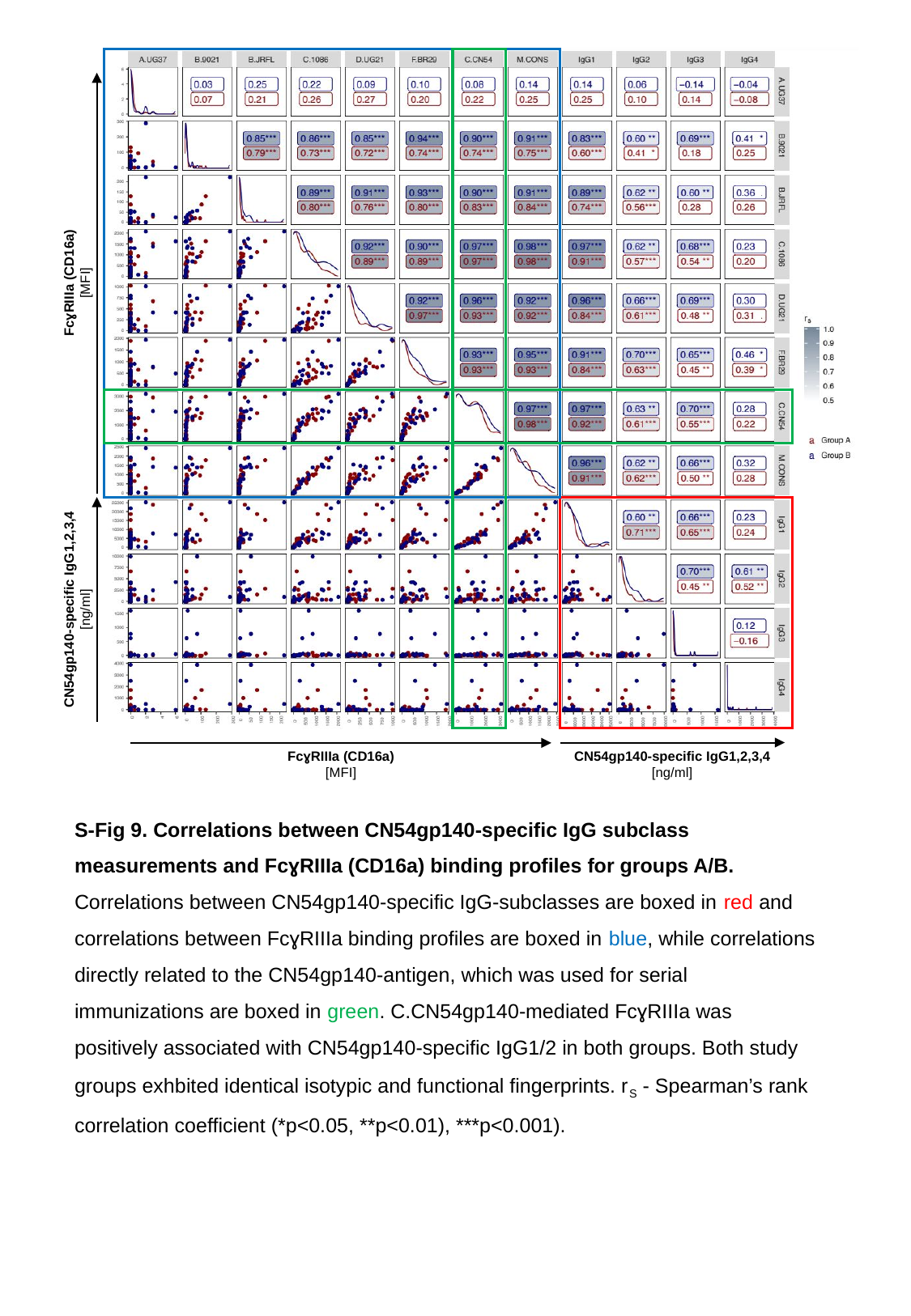

FcɣRIIIa (CD16a)
[MFI]
CN54gp140-specific IgG1,2,3,4
[ng/ml]
FcɣRIIIa (CD16a)
[MFI]
CN54gp140-specific IgG1,2,3,4
[ng/ml]
S-Fig 9. Correlations between CN54gp140-specific IgG subclass measurements and FcɣRIIIa (CD16a) binding profiles for groups A/B. Correlations between CN54gp140-specific IgG-subclasses are boxed in red and correlations between FcɣRIIIa binding profiles are boxed in blue, while correlations directly related to the CN54gp140-antigen, which was used for serial immunizations are boxed in green. C.CN54gp140-mediated FcɣRIIIa was positively associated with CN54gp140-specific IgG1/2 in both groups. Both study groups exhbited identical isotypic and functional fingerprints. rS - Spearman’s rank correlation coefficient (*p<0.05, **p<0.01), ***p<0.001).

## Slide 10
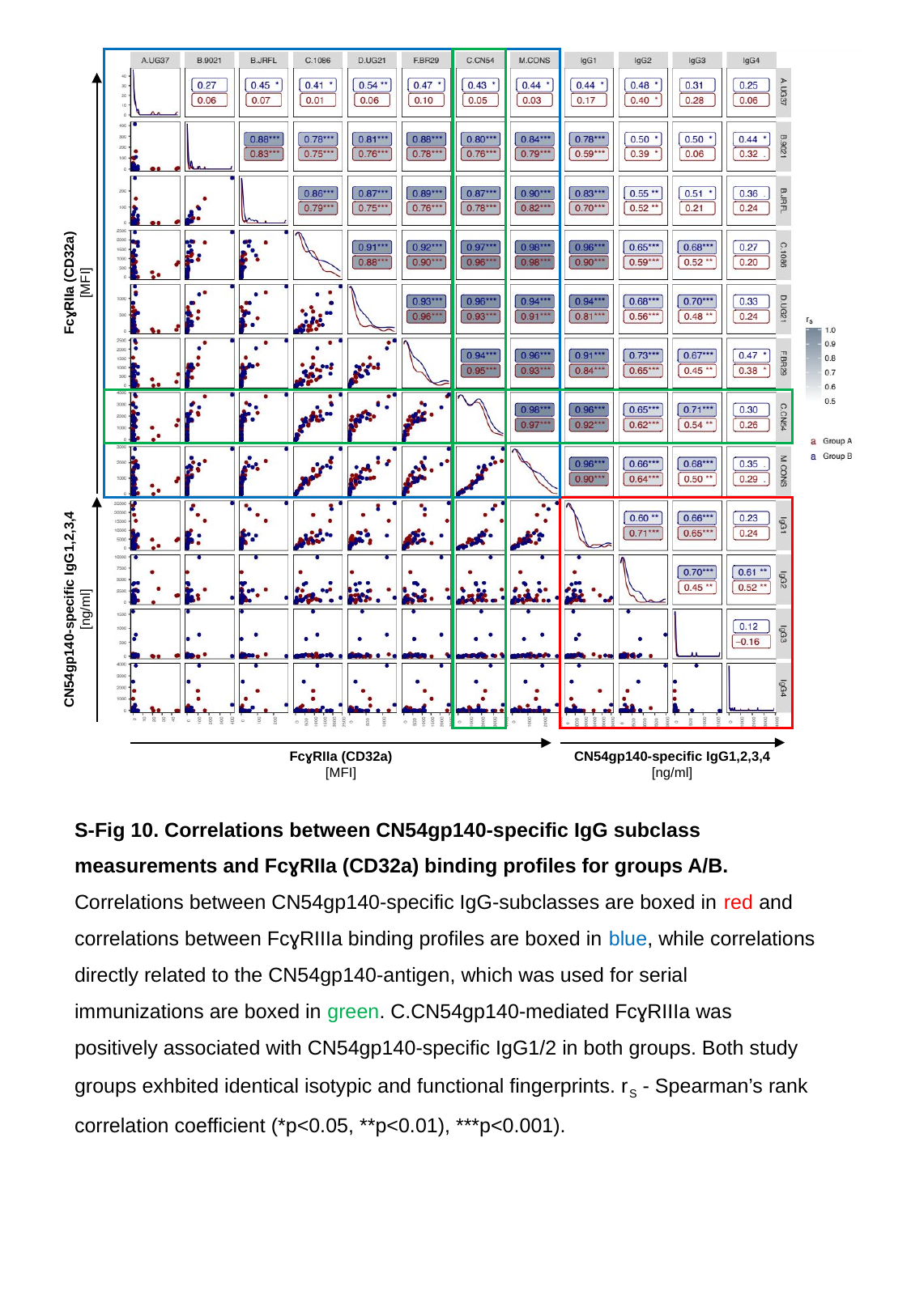

FcɣRIIa (CD32a)
[MFI]
CN54gp140-specific IgG1,2,3,4
[ng/ml]
FcɣRIIa (CD32a)
[MFI]
CN54gp140-specific IgG1,2,3,4
[ng/ml]
S-Fig 10. Correlations between CN54gp140-specific IgG subclass measurements and FcɣRIIa (CD32a) binding profiles for groups A/B. Correlations between CN54gp140-specific IgG-subclasses are boxed in red and correlations between FcɣRIIIa binding profiles are boxed in blue, while correlations directly related to the CN54gp140-antigen, which was used for serial immunizations are boxed in green. C.CN54gp140-mediated FcɣRIIIa was positively associated with CN54gp140-specific IgG1/2 in both groups. Both study groups exhbited identical isotypic and functional fingerprints. rS - Spearman’s rank correlation coefficient (*p<0.05, **p<0.01), ***p<0.001).

## Slide 11
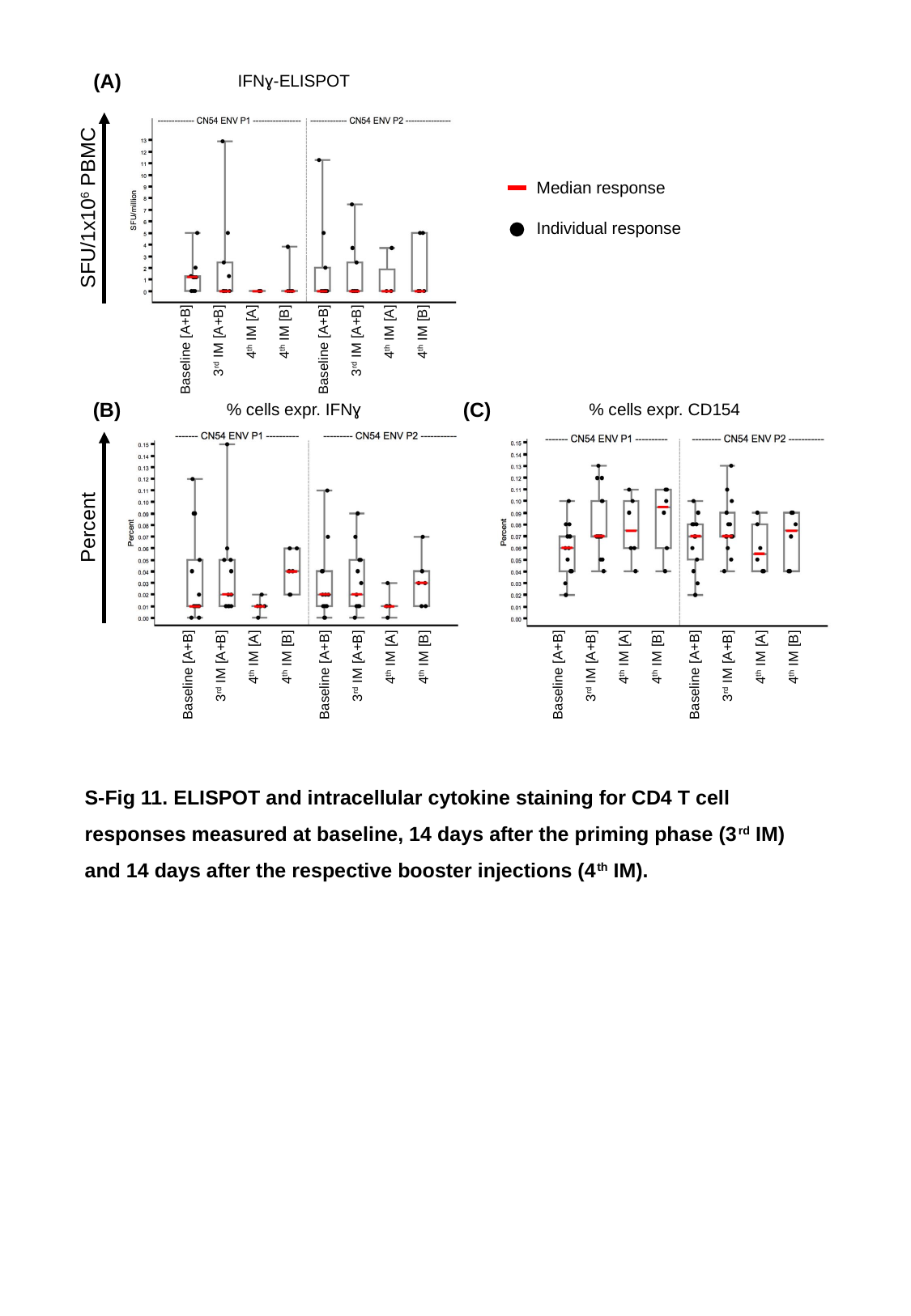

(A)
IFNɣ-ELISPOT
Median response
Individual response
SFU/1x106 PBMC
4th IM [A]
4th IM [B]
4th IM [A]
4th IM [B]
3rd IM [A+B]
3rd IM [A+B]
Baseline [A+B]
Baseline [A+B]
(B)
(C)
% cells expr. IFNɣ
% cells expr. CD154
Percent
4th IM [A]
4th IM [B]
4th IM [A]
4th IM [B]
4th IM [A]
4th IM [B]
4th IM [A]
4th IM [B]
3rd IM [A+B]
3rd IM [A+B]
3rd IM [A+B]
3rd IM [A+B]
Baseline [A+B]
Baseline [A+B]
Baseline [A+B]
Baseline [A+B]
S-Fig 11. ELISPOT and intracellular cytokine staining for CD4 T cell responses measured at baseline, 14 days after the priming phase (3rd IM) and 14 days after the respective booster injections (4th IM).

## Slide 12
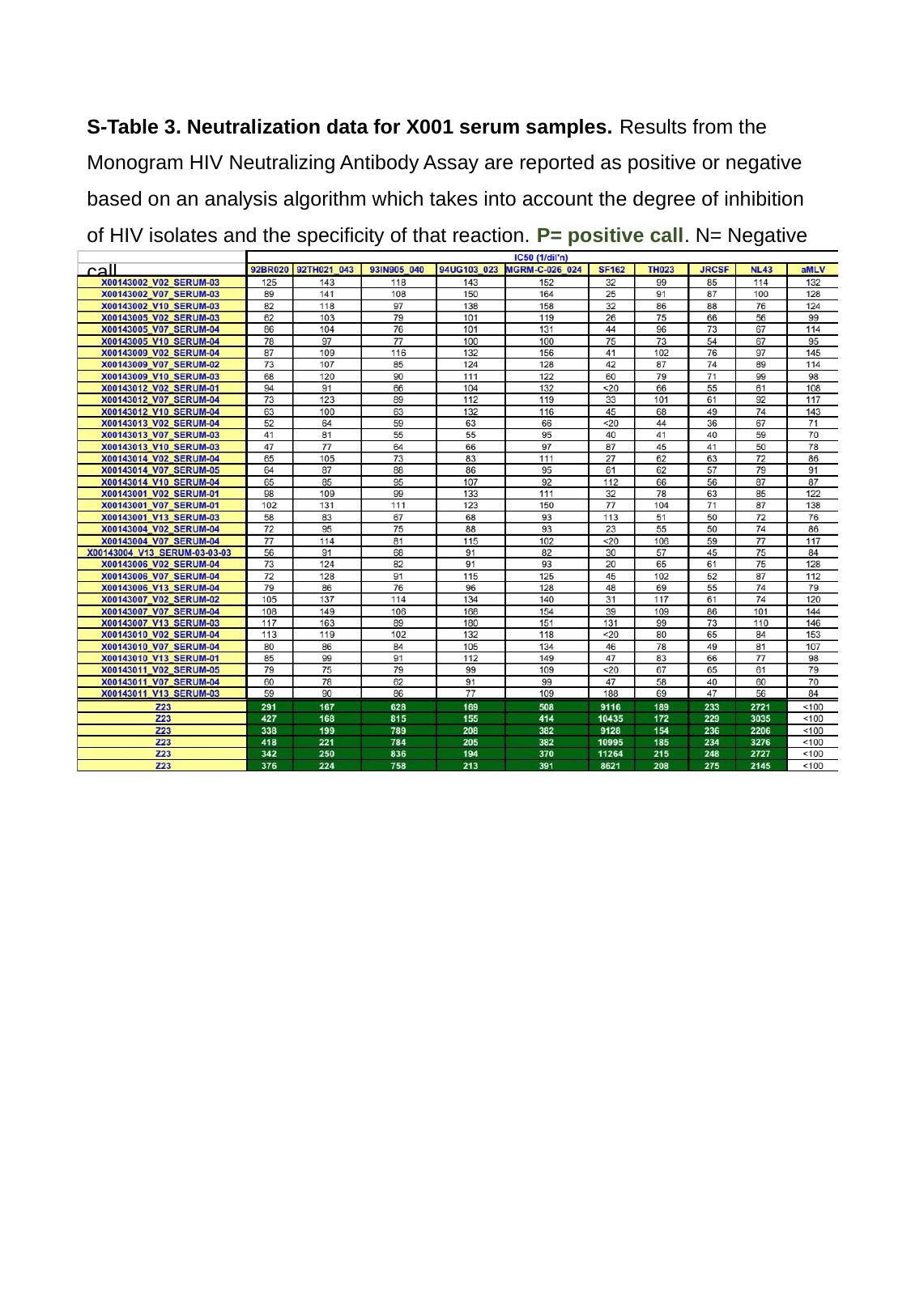

S-Table 3. Neutralization data for X001 serum samples. Results from the Monogram HIV Neutralizing Antibody Assay are reported as positive or negative based on an analysis algorithm which takes into account the degree of inhibition of HIV isolates and the specificity of that reaction. P= positive call. N= Negative call.

## Slide 13
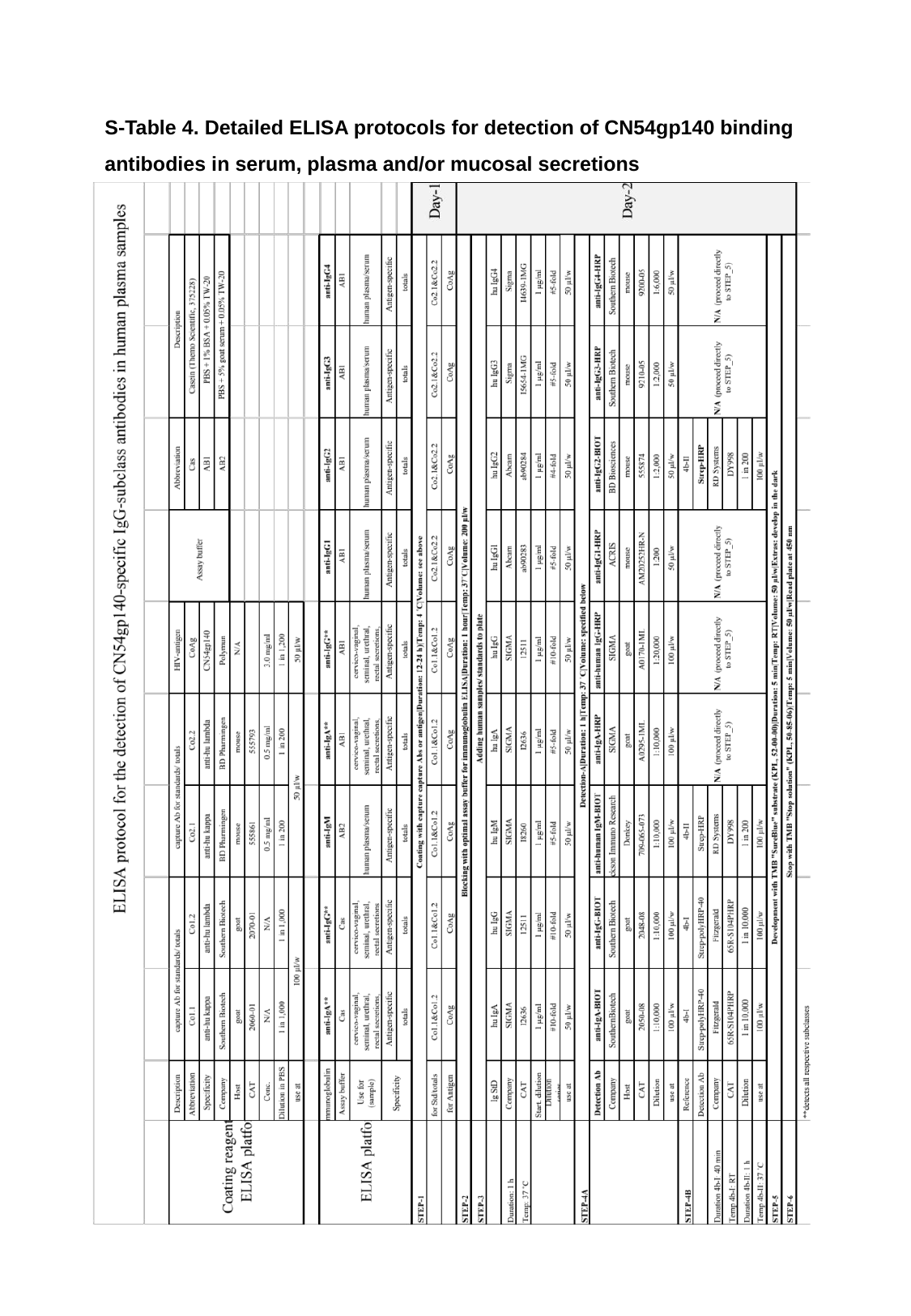

S-Table 4. Detailed ELISA protocols for detection of CN54gp140 binding antibodies in serum, plasma and/or mucosal secretions

## Slide 14
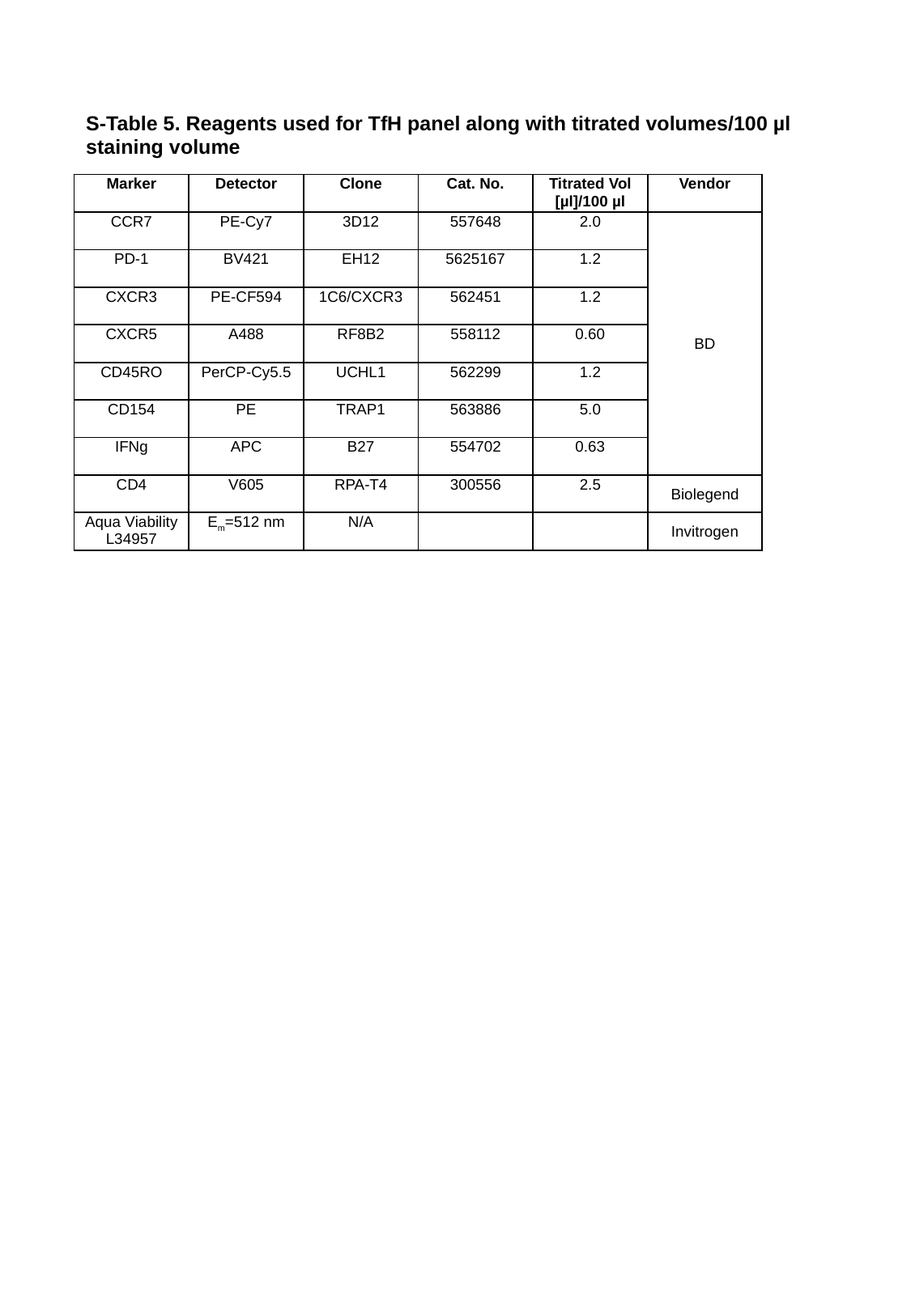

S-Table 5. Reagents used for TfH panel along with titrated volumes/100 µl staining volume
| Marker | Detector | Clone | Cat. No. | Titrated Vol [µl]/100 µl | Vendor |
| --- | --- | --- | --- | --- | --- |
| CCR7 | PE-Cy7 | 3D12 | 557648 | 2.0 | BD |
| PD-1 | BV421 | EH12 | 5625167 | 1.2 | |
| CXCR3 | PE-CF594 | 1C6/CXCR3 | 562451 | 1.2 | |
| CXCR5 | A488 | RF8B2 | 558112 | 0.60 | |
| CD45RO | PerCP-Cy5.5 | UCHL1 | 562299 | 1.2 | |
| CD154 | PE | TRAP1 | 563886 | 5.0 | |
| IFNg | APC | B27 | 554702 | 0.63 | |
| CD4 | V605 | RPA-T4 | 300556 | 2.5 | Biolegend |
| Aqua Viability L34957 | Em=512 nm | N/A | | | Invitrogen |

## Slide 15
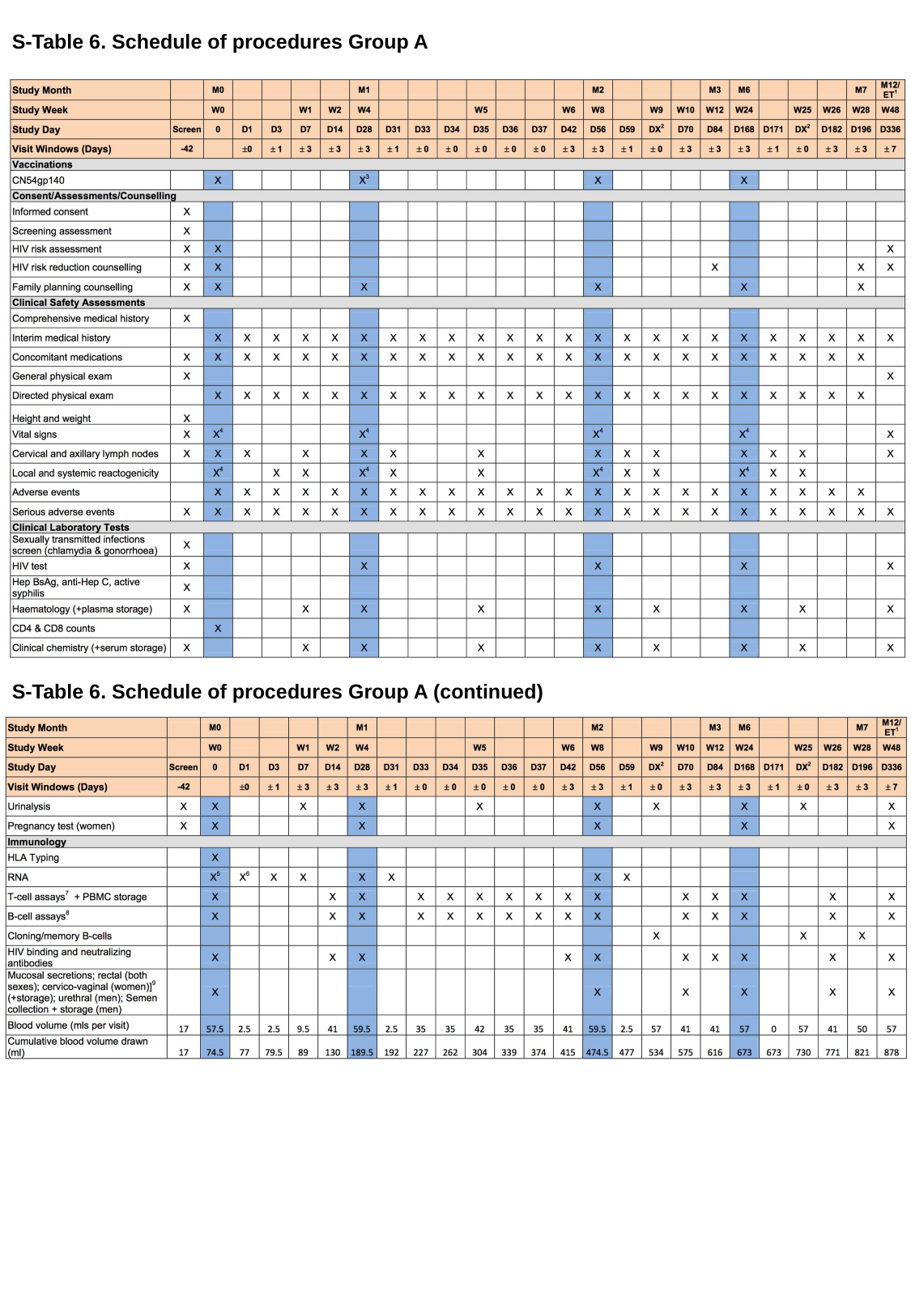

S-Table 6. Schedule of procedures Group A
S-Table 6. Schedule of procedures Group A (continued)

## Slide 16
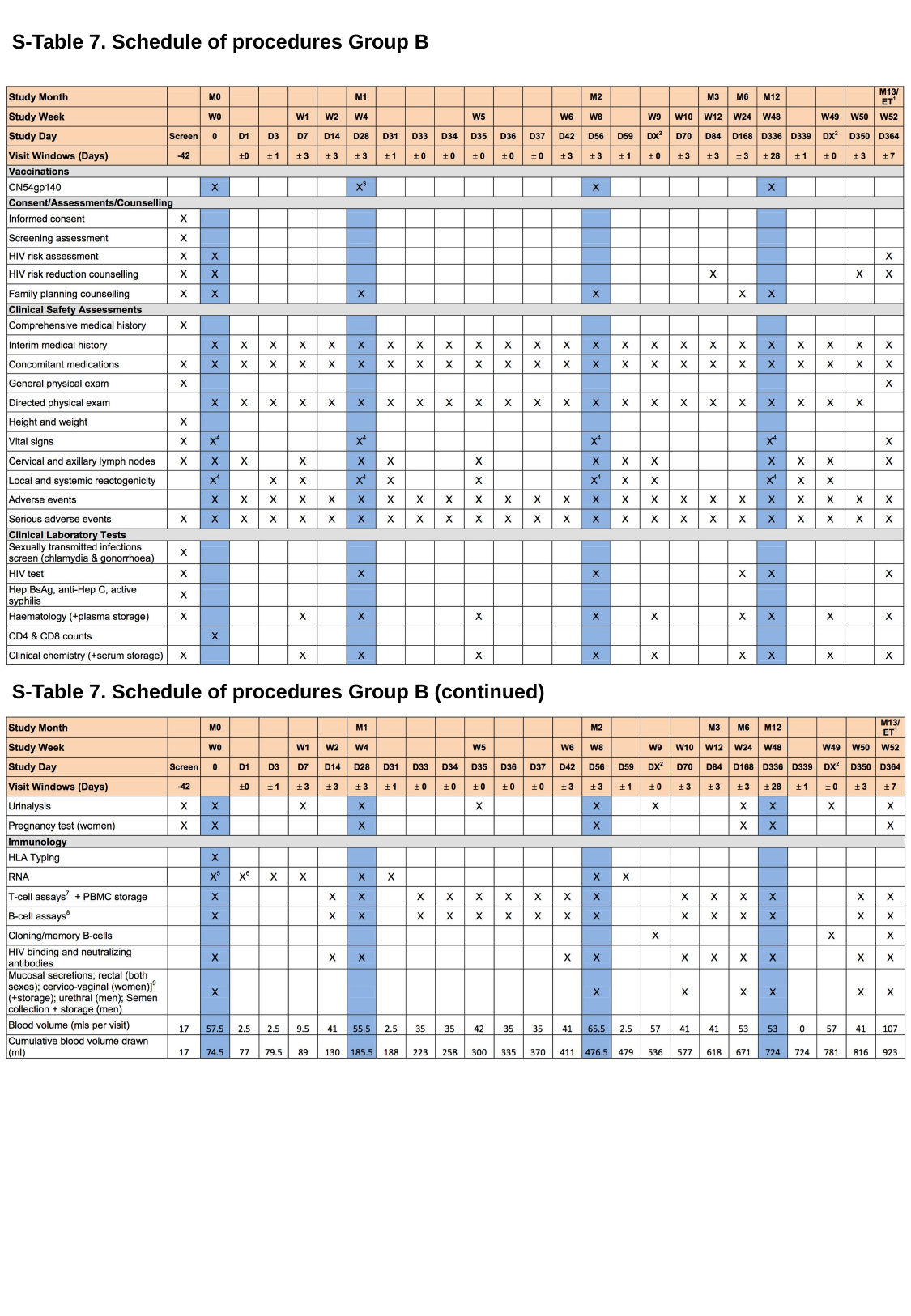

S-Table 7. Schedule of procedures Group B
S-Table 7. Schedule of procedures Group B (continued)
